# Inhibition of α-Amylase, α-Glucosidase, Pancreatic Lipase, 15-Lipooxygenase and Acetylcholinesterase Modulated by Polyphenolic Compounds, Organic Acids, and Carbohydrates of *Prunus domestica* Fruit

**DOI:** 10.3390/antiox12071380

**Published:** 2023-07-03

**Authors:** Martyna Rybak, Aneta Wojdyło

**Affiliations:** Department of Fruit, Vegetable and Nutraceutical Plant Technology, Wrocław University of Environmental and Life Sciences, 37 Chełmońskiego Street, 51-630 Wrocław, Poland

**Keywords:** *Prunus domestica*, plums, cultivars, polyphenolic identification, LCMS/QTof, antioxidant activities, ABTS^o+^, FRAP, in vitro enzyme inhibition effect, α-amylase, α-glucosidase, 15-lipoxygenase, AChE, BuChE

## Abstract

This work aimed to establish the content of phenolic compounds, carbohydrates, and organic acids and to determine their potential to inactivate α-amylase, α-glucosidase, pancreatic lipase, 15-lipoxygenase (15-LOX), acetylcholinesterase (AChE), and butyrylcholinesterase (BuChE), and antioxidant activity (ABTS^o+^ and FRAP) in 43 *Prunus domestica* cultivars. We identified 20 phenolic compounds, including, in the order of abundance, polymeric procyanidins, flavan-3-ols, phenolic acids, flavonols, and anthocyanins. The total content of phenolic compounds varied depending on the cultivar and ranged from 343.75 to 1419 mg/100 g d.w. The cultivars of Ś2, Ś11, and Ś16 accumulated the greatest amounts of polyphenols, while in cvs. Ś42, Ś35, and Ś20 polyphenols were the least abundant. The highest antioxidant potential of 7.71 (ABTS^o+^) and 13.28 (FRAP) mmoL Trolox/100 g d.w. was confirmed for cv. Ś11. *P. domestica* fruits showed inhibitory activity toward α-amylase (2.63–61.53), α-glucosidase (0.19–24.07), pancreatic lipase (0.50–8.20), and lipoxygenase (15-LOX; 4.19–32.67), expressed as IC_50_ (mg/mL). The anti-AChE effect was stronger than the anti-BuChE one. Cv. Ś3 did not inhibit AChE activity, while cv. Ś35 did not inhibit BuChE. Thanks to the abundance of biologically active compounds, *P. domestica* offers several health-promoting benefits and may prevent many diseases. For these reasons, they are worth introducing into a daily diet.

## 1. Introduction

Over 400 species in the *Prunus* genus belong to the *Rosacae* family, but only 89 are listed in the Genetic Resources Information System [1]. The main representatives of the *Prunus* genus are plums, cherries, peaches, apricots, and almonds [2]. Plum (*Prunus* L.) fruits have gained importance in recent years, as they constitute a considerable share of fruit production in Poland and Europe. The fruits are valued for sensory reasons (taste and smell) and technological properties (attractive and desirable products). The most commonly grown cultivars are European plum (*Prunus domestica* L.) and Japanese plum (*Prunus salicina*) [1]. *P. domestica* fruits come in different skin and flesh colours, which, depending on the cultivar, range from dark purple-red, red to yellow or yellowish-green.

The fruits can be divided into early (‘Królowa Wiktoria’, ‘Kirka’), moderately early (‘Renkloda’, ‘Węgierka’), and late (‘Anna Spath’, ‘Stanley’) [3]. A wide variety of *P. domestica* cultivars, including late-ripening ones, considerably prolongs their availability on the market, even until October, in the climatic conditions of Europe. Therefore, *P. domestica* fruits are the second, after peaches and nectarines, the most often produced stone fruits worldwide. Their cultivation area is 2,700,000 ha, and their total annual production is about 12,600,000 tones [4]. With such abundance, *P. domestica* fruit can be eaten in many ways, either as fresh fruit or as a processed product. *P. domestica* fruits are used for dried [5], candied or frozen products, jams, mousses, compotes, juices, and powders [6]. With the current “zero to waste” trend, seeds, which account for 10% of the total fruit weight, are also utilized. They are a source of desirable substances for the cosmetics and pharmaceutical industry but less often for the food industry [7].

*P. domestica* fruits are a rich source of health-promoting substances and nutrients, the content of which varies depending on the cultivar. Due to this variety, the fruits differ significantly in their chemical composition, including the content of simple sugars, organic acids, dietary fiber, minerals, such as K, P, and Ca, vitamins C, A, or those of the B group [5]. *P. domestica* fruits have a high content of water (88%) and low fats, which makes them low in calories (about 45 kcal/100 g) [7]. Regular consumption of dried *P. domestica* fruits aids in anticancer, antidiabetes, and antiobesity prevention and improves the functioning of the digestive system [8]. Compared with other fruits, *P. domestica* ones are characterized by a significant antioxidant potential, which results from their high content of bioactive compounds, including polyphenols. The most important compounds of *P. domestica* fruit include anthocyanins, flavonol derivatives, and phenolic acids, such as chlorogenic and neochlorogenic acids [1].

*P. domestica* fruits are valued for their positive effect on the human body. Flavonoids and phenolic acids of *P. domestica* fruits have strong antioxidant, anti-inflammatory, antidiabetic, and anticancer properties. Their high content of anthocyanins and flavonols plays an important role in neuroprotection and prevention of cardiovascular diseases [2]. Thanks to the biologically active compounds, the fruits of the genus *Prunus* show anticarcinogenic properties. Research conducted by Fang and colleagues [9] proved that apigenin contained in the fruit inhibited the expression of hypoxia-inducible factor 1 (HIF-1) and vascular endothelial growth factor (VEGF) in human ovarian cancer cells. In addition, apigenin inhibited tumorigenesis, as measured by the Matrigel test and the chorioallantoic membrane test (CAM test) [2]. A systematic review by Igwe and Charlton [10] revealed that consumption of *P. domestica and P. salicyna* fruits improved cognitive function and bone health parameters and reduced cardiovascular risk factors. Undoubtedly, these health-promoting qualities depend on the presence of biologically active substances.

This work aimed to characterize the qualitative and quantitative content of polyphenolic compounds (flavan-3-ols, flavonols, anthocyanins, and phenolic acids) and to determine the antioxidant (ABTS^o+^, FRAP), antidiabetic (inhibition of α-amylase and α-glucosidase), antiobesity (inhibition of pancreatic lipase), and antidementia (inhibition of acetylcholin- and butyrylcholinesterase) activity of the fruits of 43 *P. domestica* cultivars grown in the climatic region of Poland. We also determined their content of sugars and organic acids to establish possible relationships based on the principal component analysis (PCA) and agglomeration hierarchical clustering (AHC).

## 2. Methods and Materials

### 2.1. Plant Material

The examined plant material included 43 *Prunus domestica* cultivars: ‘Valor’ (Ś1), ‘Cacanska najbolja’ (Ś2), ‘Top’ (Ś3), ‘Cacanska rana’ (Ś4), ‘Silvia’ (Ś5), ‘Verity’ (Ś6), ‘Stanley’ (Ś7), ‘Pitestean’ (Ś8), ‘Magna glaucia’ (Ś9), ‘SL3′ (Ś10), ‘Sanctus Hubertus’ (Ś11), ‘Cacanska lepotica’ (Ś12), ‘SL 13′ (Ś13), ‘Węgierka łowicka’ (Ś14), ‘Fryga’ (Ś15), ‘Tolar’ (Ś16), ‘Opal’ (Ś17), ‘Vision’ (Ś18), ‘Nectavit’ (Ś19), ‘Amers’ (Ś20), ‘Jojo’ (Ś21), ‘Hanita’ (Ś22), ‘Empress’ (Ś23), ‘Węgierka włoska’ (Ś24), ‘Węgierka zwykła’ (Ś25), ‘Valjevka’ (Ś26), ‘Bluefre’ (Ś27), ‘Węgierka wczesna’ (Ś28), ‘Węgierka dąbrowicka’ (Ś29), ‘Węgierka wangenheima’ (Ś30), ‘Diana’ (Ś31), ‘Królowa Wiktoria’ (Ś32), ‘Erliblue’ (Ś33), ‘Katinka’ (Ś34), ‘Czernowitzer’ (Ś35), ‘Elena’ (Ś36), ‘Anna Spath’ (Ś37), ‘Oneida’ (Ś38), ‘Brzoskwiniowa’ (Ś39), ‘Mirabelka z Nancy’ (Ś40), ‘Renkloda Ulena’ (Ś41), ‘Herman’ (Ś42), and ‘Ruth Gerstetter’ (Ś43). The material was obtained from the Research Center for Cultivar Testing in Zybiszów near Wrocław.

### 2.2. Sample Preparation

Each cultivar was represented by at least 10 *P. domestica* fruits randomly collected from a single tree. After pitting, the fruits were sliced in liquid nitrogen and lyophilized for 24 h (Christ Alpha 1–4 LSC; Martin Christ Gefriertrocknungsanlagen GmbH; Osterode am Harz, Germany). The homogeneous powder was obtained by grinding the *P. domestica* fruits in a closed laboratory mill (IKA A.11; Staufen, Germany). The powder was protected against unfavorable external factors to avoid hydration for 48 h without access to light.

### 2.3. Identification and Quantification of Polyphenolic Compounds

The content of polyphenolic compounds was determined by Ultra Performance Liquid Chromatography type, Acquity Ultra Performance LC by Waters. The samples (about 1 g) were extracted with 9 mL of a mixture containing methanol:H_2_O:ascorbic acid:98% of acidic acid (30:67:2:1, *v*/*v*/*w*/*v*). The extraction was performed twice by incubating the samples for 20 min using sonication (Sonic 6D, Polsonic, Warsaw, Poland), with occasional shaking. Then the suspension was centrifuged at 19,000× *g* for 10 min (MPW-350R; Warsaw, Poland), and the supernatant was filtered through a hydrophilic PTFE membrane (0.2 µm; Millex Samplicity Filter; Merck, Darmstadt, Germany), and used for further analysis. All the extractions were performed in triplicate.

Qualitative (LC-MS-Q/TOF) and quantitative (UPLC-PDA) analysis of polyphenols (flavan-3-ols at 280 nm, flavonols at 360 nm, phenolic acids at 320 nm, and anthocyanins at 520 nm) was performed as previously described by Wojdyło, Nowicka, Bąbelewski [11] and Wojdyło, Carbonell-Barrachina, Legua and Hernández [12]. Individual polyphenols were separated on an ACQUITY UPLC BEH C18 column (1.7 µm, 2.1 × 100 mm; Waters Corporation, Milford, CT, USA) at 30 °C. The samples (5 µL) were injected, and elution was completed after 15 min by gradient and isocratic sequence at a flow rate of 0.42 mL/min. The program started with a gradient elution with 99 to 65% solvent A (0–12 min), followed by a reduction of solvent A to 0% for the conditioning column (12.5–13.5 min), after which the gradient returned to its original composition (99% A) at 14 min. Then the column was equilibrated for 1 min, and at min 15, another analysis began. The mobile phase comprised solvent A (2% formic acid in H_2_O, *v*/*v*) and solvent B (100% acetonitrile). All measurements were repeated three times. The content of polyphenolic compounds was expressed in mg/100 g of dry weight (d.w.).

### 2.4. Analysis of Polymeric Procyanidins by Phloroglucinolysis

The analysis of the polymer fractions of procyanidins was carried out using the Ultra Performance Liquid Chromatography system type, Acquity Ultra Performance LC, by Waters. The analysis was performed as previously described by Wojdyło and Oszmiański [13]. Phloroglucinolysis products were separated on a BEH Shield C18 RP column (1.7 µm, 2.1 × 100 mm; Waters Corporation, Milford, CT, USA) with solvent A (2.5% acetic acid in H_2_O) and solvent B (100% acetonitrile). The cycle was set with the following gradient: 0–1.0 min, 2% B; 1.00–2.5 min, 2–3% B; 2.5–3.5 min, 3–10% B; 3.5–5.5 min, 10–15% B; 5.5–7.5 min, 100% B, followed by washing and conditioning of the column. The flow rate was 0.45 mL/min, the injection volume was 5 µL, and the column temperature was 30 °C. Fluorescence was recorded at the excitation wavelength of 278 nm and the emission wavelength of 360 nm. Calibration curves for quantification were made with procyanidin B2, (+)-catechin, and (-)-epicatechin. The average degree of polymerization was calculated as the molar ratio of all flavan-3-ol units to terminal units of (-)-epicatechin and (+)-catechin. All the samples were analyzed in triplicate, and the results were expressed in mg per 100 g d.w.

### 2.5. Organic Acid and Carbohydrate Content

The analysis of organic acids and carbohydrate was performed as described previously by Wojdyło et al. [14], using HPLC-PDA (Waters Co.; Milford, CT, USA) and HPLC-ELSD (PL-ELS 1000; Merck; Hitachi, Japan), respectively. The sample (approximately 3 g of fruit) was mixed with distilled water, exposed to ultrasounds (Sonic 6D; Polsonic, Warsaw, Poland) for 15 min, heated at 90–100 °C for 30 min, and finally centrifuged (MPW-55; Warsaw, Poland) at 12,000× *g* for 10 min at 4 °C. The supernatant (2.5 mL) was injected into a Sep-Pak C-18 cartridge (1 g, Millipore Waters, Milford, MA, USA) and eluted with H_2_O into Eppendorf tubes. Before analysis, the extract was filtered through a hydrophilic PTFE membrane (0.20 µm; Millex Simplicity filters; Merck, Germany). The organic acids were analyzed on Polymex IEX H column (8 μm, 250 × 8 mm, Watrex; Prague, Czech Republic) using isocratic elution with 0.9 M sulfuric acid in H_2_O for 20 min. The carbohydrates were analyzed on Alltech^®^ Prevail^TM^ Carbohydrate ES HPLC Column-W 250 × 4.6 mm, 5 µm (Columbia, MD, USA) using isocratic elution with 70% acetonitrile in H_2_O for 20 min. The results were expressed in g per 100 g of d.w.

### 2.6. Analysis of Biological Activity

All analyses were made using a multi-mode microplate reader SynergyTM H1 (BioTek, Winooski, VT, USA) in three repetitions. The antioxidant activity of ABTS^o+^ and FRAP was expressed in mmoL Trolox per 100 g. Other results were expressed as the sample capable of reducing the enzyme activity by 50% (IC_50_) in mg/mL.

#### 2.6.1. Analysis of Antioxidant Activities of ABTS^o+^ and FRAP

Antioxidant properties were assessed using the ABTS^o+^ method, which determines the ability to reduce the ABTS^o+^ cation radical, and the FRAP method, in which Fe^3+^ is reduced to Fe^2+^. The samples for the analysis were prepared as described by Wojdyło et al. [11]. The fruit powder (about 0.5 g) was mixed with 5 mL of methanol:H_2_O:HCl (79:20:1; *v*/*v*/*v*), exposed to ultrasounds at 20 °C for 15 min and left for 24 h at 4 °C. Then, the extract was again exposed to ultrasounds for 15 min and centrifuged for 15 min at 15,000× *g*. In triplicate, all measurements were performed using a PC UV-2401 spectrophotometer (Shimadzu, Kyoto, Japan). The antioxidant activity of ABTS^o+^ and FRAP was expressed in mmol Trolox per 100 g d.w.

#### 2.6.2. Inhibition of α-Amylase, α-Glucosidase, and Pancreatic Lipase

The inhibitory effect on the activity of α-amylase and α-glucosidase (antidiabetic activity), and pancreatic lipase (antiobesity activity) of *P. domestica* fruits was determined according to the procedure described before by Wojdyło et al. [15,16]. The inhibition of α-amylase activity by the *P. domestica* extracts was evaluated using the ability of α-amylase to hydrolyze α-1,4-glycosidic bonds. The hydrolysis causes gradual cleavage of starch chains and results in a color reaction of iodine with KJ. Depending on the degree of starch degradation, the colour is dark blue to violet after incubation at 37 °C and shows maximum absorption at 600 nm. Inhibition of α-glucosidase activity by the *P. domestica* extracts was evaluated based on the interaction of α-glucosidase with PNPG (4-nitrophenyl-α-D-glucopyranose). This reaction in an alkaline environment yields glucose and p-α-nitrophenol (PNG). The latter is yellow and shows a maximum absorbance of 405 nm. The stronger the enzyme inhibition capacity of the tested extracts, the less p-α-nitrophenol is released from PNPG due to enzymatic hydrolysis.

Reference samples and positive control were prepared with a buffer instead of the enzymes and acarbose.

Inhibition of pancreatic lipase by the *P. domestica* extracts was evaluated based on the enzyme activity mediating the formation of *p*-nitrophenol from *p*-nitrophenol acetate at 37 °C. The reaction product shows maximum absorbance at 400 nm. Reference samples and positive control were prepared with a buffer instead of the enzyme and orlistat.

#### 2.6.3. Inhibition of 15-Lipoxygenase

Inhibition of 15-lipoxygenase activity was determined as described by Wojdyło et al. [17]. The inhibitory properties of the *P. domestica* extracts were assessed based on the formation of conjugated double bonds in linoleic acid hydroperoxide during the reaction carried out at 37 °C for 20 min. The product showed a maximum absorbance of 210 nm [15]. In the reference samples, the enzyme was replaced with Tris-HCl buffer. The results were expressed as IC_50_ values.

#### 2.6.4. Inhibition of Acetylcholinesterase (AChE) and Butyrylcholinesterase (BuChE)

Inhibition of cholinesterase was assessed using the acetylcholinesterase (AChE) and butylcholinesterase (BuChE) methods described before by Wojdyło et al. [17]. The reaction mixture consisted of a sample of *P. domestica* extract, Tris-HCl buffer (pH 8.0), acetylthiocholine iodide or S-butylthiocholine iodide and 5,5′-dithiobis(2-nitrobenzoic acid). After incubation at 37 °C for 10 min, AChE or BuChE solution was added. The absorbance was measured after 15 min at 412 nm. The results were expressed as IC_50_ (mg/mL). All assays were performed in triplicate with a PC UV-2401 spectrophotometer (Shimadzu, Kyoto, Japan).

### 2.7. Statistical Analysis

The statistical analysis was performed with the Statistica package, version 15.03 (StatSoft, Kraków, Poland). Significant differences (*p* ≤ 0.05) between mean responses were assessed by one-way ANOVA with the Duncan test. Principal component analysis (PCA) was performed using XLSTAT Statistical Software for Microsoft Excel 2017 (Microsoft Corp., Redmond, WA, USA).

## 3. Results and Discussion

### 3.1. Content of Carbohydrates and Organic Acids

Organic acids and carbohydrates contribute significantly to the sensory desirability of fruits, conferring their pleasant taste and aroma. The sugar-to-organic acid ratio is an important quality indicator. The higher the ratio, the more attractive the fruits and the products of their processing [18]. The composition and content of sugars and organic acids in fruit depend mainly on the cultivar. However, environmental factors and growing conditions may also affect their total content [19]. From the technological perspective, acids affect the gelling properties of pectin. They are also less susceptible to changes during storage and processing than other fruit components, such as flavor and aroma compounds or pigments [18].

Carbohydrates content in *P. domestica* cultivars was highly variable and ranged from 15.51 to 5.49 g/100 g d.w. (Table 1). The main saccharides identified were sucrose and fructose, followed by glucose and sorbitol. Another study showed the dominance of fructose and glucose in the total carbohydrate pool of *Prunus domestica* L. fruit, while sucrose and sorbitol were detected at low amounts [18]. The highest total carbohydrates content was identified in the cultivars Ś25, Ś30, Ś26, Ś11, Ś38, Ś40, and Ś43 (>13 g/100 g d.w.), and the lowest in cvs. Ś32, Ś17, Ś35, Ś27, Ś42, Ś29, Ś15, and Ś21 (<8 g/100 g d.w.). Wu et al. [20] analyzed fruits of various *Prunus persica* L. *Batsch* cultivars and found sucrose to be the most abundant carbohydrate in all cultivars. Also, all the cultivars had a higher content of fructose than glucose.

Depending on the cultivar, *P. domestica* fruits are much richer in sugars than cherries (16.33 and 9.09 mg/100 g, respectively). A comparison of *P. domestica* fruits with apple [21] showed the same or higher content of sugars in *P. domestica*. Finally, *P. domestica* fruits contained less sugar than peaches [22].

The content of organic acids in the analyzed *P. domestica* cultivars varied and ranged from 4.33 to 1.34 g/100 g d.w. (Table 1). The most common organic acid was malic acid, quinic, and oxalic acid. For the other acids, the order of abundance was as follows: succinic, formic, and citric acid, and they were detected only in trace amounts.

The highest total content of organic acids was determined for the cultivars Ś42, Ś27, Ś23, Ś18, Ś4, Ś13, Ś41, Ś35, Ś7, Ś8, and Ś24 (>2.5 g/100 g d.w.), and the lowest for cvs. Ś12, Ś25, Ś34, Ś31, Ś28, and S10 (<1.6 g/100 g d.w.). The content of malic acid, with the greatest share in total acidity, was consistent with previous data published by Tomić et al. [18].

According to Colaric et al. [23], the ratio of sugars to organic acids, and above all, the content of citric and shikimic acid, have a significant and decisive impact on the perception of sweetness and sourness and thus play an essential role in conferring the taste of the fruit. This ratio for the tested *P. domestica* fruits ranged widely from 1.47 to 11.96. The cultivars Ś25 (11.96), Ś12 (9.08), Ś26 (8.82), Ś34 (8.57), and Ś10 (8.08) were characterized by the highest sugar-to-organic acid ratio. The ratio was the lowest in the cultivars with the highest content of organic acids, that is, Ś27 (1.47), Ś42 (1.53), Ś35 (2.25), and Ś23 (2.38).

The fruits most popular among consumers should have a ten times high sugar to organic acid ratio [24].

### 3.2. Identification and Quantification of Polyphenolic Compounds in P. domestica Fruits

Polyphenols are phenolic compounds synthesized in plants as by-products or secondary metabolites. They provide significant health benefits in human nutrition. For example, they reduce the risk of developing chronic diseases, and have antioxidant, anti-inflammatory, anticancer, antiallergic, antihypertensive, and antiviral properties [25]. In the fruits of the investigated *P. domestica* cultivars, LC-MS Qtof analysis confirmed the presence of 19 phenolic compounds belonging to four groups: phenolic acids (neochlorogenic, cryptochlorogenic, chlorogenic, 3-caffeoylshikimic, and 3-feruloylquinic acid), flavonols (quercetin of -pentoside-hexoside, -3-*O*-galactoside, -3-*O*-glucoside, -3-*O*-rutinoside, -3-*O*-arabinoside, 3-*O*-rhamnoside, 3-*O*-penthoside-rhamnoside), flavan-3-ols (procyanidin B1 and B3, (+)-catechin), and anthocyanins (cyanidin-3-*O*-galactoside, -3-*O*-glucoside, -3-*O*-rutinoside, and peonidin-3-*O*-glucoside) (Table 2).

The content of phenolic compounds in *P. domestica* fruits has been studied by some researchers [18,26], but these studies were limited to several or single cultivars different from those analyzed in our work. In this study, we quantified the concentration of anthocyanins, phenolic acids, flavan-3-ols, and flavonols (Table 3). The average content of polyphenols ranged from 343.75 to 1419.14 mg/100 g d.w. These values indicated significant differences between the cultivars regarding the total content of polyphenols. The highest total content of polyphenolic compounds was determined for cvs. Ś2, Ś9, Ś11, Ś12, Ś16, Ś17, Ś18, Ś19, Ś21, Ś29, Ś33 and Ś43 (>1000 mg/100 g d.w.), and the lowest for cvs. Ś7, Ś20, Ś35, and Ś42 (<500 mg/100 g d.w.).

Flavan-3-ols were the main group of polyphenols (16.70–83.79%). Phenolic acids constituted next the main group of polyphenolic compounds, accounting for 10.47% to 69.08% of the total phenolic compounds. The other most abundant groups were flavonols (0.82% to 16.08%). Regardless of the cultivar, the least abundant group were anthocyanins (0.35% to 6.63%). The main reason for significant differences in the content of polyphenolic compounds was the cultivar, and the other factors were related to the method of cultivation, climatic conditions of a growing season, and the degree of fruit maturity [27].

Flavan-3-ols belong to flavonoids and play an important role in plant antioxidant activity, including scavenging free radicals, chelation of transition metals, and mediation and inhibiting enzymes. These compounds confer multiple beneficial health-promoting properties, acting as anticancer, antibacterial, antiviral, and cardio- and neuroprotective agents while protecting against cardiovascular diseases [18]. The number-3-ols in the fruits of the investigated *P. domestica* cultivars ranged from 11.69 to 124.17 mg/100 g d.w. (Table 2). Their total highest content was determined in cvs. Ś2, (>1000 mg/100 g d.w.), and the lowest in cvs. Ś7, Ś15, Ś22, Ś26, Ś31, Ś32, Ś35, Ś36, Ś37, Ś40, Ś42, and Ś42 (<300 mg/100 g d.w.). Three flavan-3-ols were detected in the analyzed *P. domestica* cultivars. Polymeric procyanidin was the most abundant flavanol (77.31–97.27%) in all tested cultivars. Procyanidin B1 was the second most abundant flavanol (>13.96%), followed by (+)-catechin (>3.26%) and finally by procyanidin B3 (>2.73%). The remaining detected flavan-3-ol derivatives accounted for up to 17.33% of the total flavanols. They were the most abundant in cv. Ś2 fruits (1114.47 mg/100 g d.w.), and the least abundant in cv. Ś41 (163.49 mg/100 g d.w.).

Procyanidins are oligomeric compounds formed from (+/−)-(epi)catechin molecules [12,15,28]. They show a protective effect, particularly in preventing chronic metabolic diseases, by minimizing cell damage caused by oxidative stress. They show potent antioxidant properties and can scavenge free radicals and nitrogen forms [29]. Regular consumption of flavan-3-ols alleviates the pathological features of Alzheimer’s disease, improves cognitive functions, and modulates synaptic plasticity [28]. Procyanidins are claimed to inhibit gastrointestinal lipase, thereby reducing plasma triglyceride levels [30].

Phenolic acids have a high antioxidant capacity and exhibit antibacterial, antiviral, anticancer and anti-inflammatory properties [25]. The number of phenolic acids in the fruits of the investigated *P. domestica* cultivars ranged from 83.20 to 804.77 mg/100 g d.w. The highest total content of phenolic acids was determined in cvs. Ś16, Ś17, Ś18, Ś19, Ś11, Ś40, Ś22, and Ś29 (>500 mg/100 g d.w.), and the lowest in cvs. Ś42, Ś20, Ś7, Ś12, Ś28, Ś35, Ś21, and Ś25 (<200 mg/100 g d.w.). We identified five phenolic acids, among which neochlorogenic acid accounted for 78.17% of their total amount. The highest content of neochlorogenic acid was determined in cvs. Ś17, Ś16, Ś11, Ś18, Ś19, Ś29, Ś22, and Ś40 (>400 mg/100 g d.w.), and the lowest in cvs. Ś20, Ś7, Ś42, and Ś12 (<100 mg/100 g d.w.). Chlorogenic acid constituted 10.5% of the total amount of phenolic acids, with the highest values in cvs. Ś16, Ś37, Ś33, Ś18, Ś32, and Ś31 (>60 mg/100 g d.w.), and the lowest in cvs. Ś27, Ś28, Ś21, and Ś42 (<10 mg/100 g d.w.). Another identified acid was cryptochlorogenic acid, which accounted for 3.64% of all phenolic acids. It was the most abundant in cvs. Ś17, Ś16, Ś11, and Ś19 (>30 mg/100 g d.w.), and the least abundant in cvs. Ś4, Ś20, Ś27, Ś42, Ś7, Ś8, Ś12, and Ś35 (<5 mg/100 g d.w.). 3-Feruloylquinic acid constituted 1.84% of the total phenolic acids, with the highest values in cvs. Ś16, Ś33, Ś19, Ś37, Ś22, Ś14, and Ś1 (>10 mg/100 g d.w.), and the lowest in cvs. Ś41, Ś5, Ś4, Ś27, Ś40, Ś15, Ś6, Ś24, and Ś35 (<3 mg/100 g d.w.). The last identified acid was 3-caffeoyl shikimic acid, which accounts for 1.06% of the total phenolic acids. The cultivars with the highest content were Ś16, Ś31, Ś18, Ś29, and Ś17 (>6 mg/100 g d.w.), and those the least abundant in it were Ś20, Ś23, Ś25, Ś37, Ś42, and Ś28 (<1 mg/100 g d.w.). The remaining phenolic acids were pooled together, accounting for 5.17% of the total phenolic acids. In other studies [31,32], neochlorogenic acid was also shown to be the dominant compound, closely followed by chlorogenic acid at lower concentrations.

Phenolic acids usually occur in plants in a bound form of esters and glycosides. They are extremely important for plants, as they actively defend the living tissues against injuries, infections, or insolation. Phenolic acids are characterized by antioxidant activity. They regulate seed germination and plant growth. Esterified derivatives of caffeic acid that is, neochlorogenic and chlorogenic acids, have strong antioxidant, antimutagenic, and anticancer properties and regulate carbohydrate metabolism by lowering the level of glucose in the human body [30]. Research conducted by Navarro-Orcajada et al. [30] revealed anti-inflammatory, hepatoprotective, antimicrobial, cardioprotective, and neuroprotective effects, and Zhao et al. [28] also reported their antihypertensive properties.

Flavonols are the most common biologically active compounds in plants and have several beneficial properties. The flavonols in the investigated *P. domestica* cultivars ranged from 7.6 to 128.77 mg/100 g d.w. (Table 2). Their highest total content was determined for cvs. Ś32, Ś18, Ś39, Ś31, and Ś37 (>75 mg/100 g d.w.), and the lowest for cvs. Ś28, Ś27, Ś24, Ś21, Ś8, Ś42, Ś4 (<25 mg/100 g d.w.). In the analyzed *P. domestica* fruit samples, we identified seven flavonols, of which quercetin-3-*O*-galactoside, -3-*O*-glucoside, -3-*O*-rutinoside, and -3-*O*-arabinoside were present in all cultivars. In the total pool of flavonols, quercetin-3-*O*-rutinoside accounted for 34.18%, quercetin-3-*O*-galactoside for 21.82%, quercetin-3-*O*-glucoside for 13.18%, quercetin-3-*O*-arabinoside for 10.6%, quercetin-penthoside-rhamnoside for 4.72%, quercetin-pentoside-hexoside for 3.77%, and quercetin-rhamnoside for 3.33%. The remaining compounds from this group accounted for 8.2% of all flavonols (Table 2). Another study by Popović et al. [31] also identified quercetin-3-*O*-rutinoside as a dominant flavonol in *Prunus*.

Quercetin, a compound commonly found in plants, shows many biological activities [33]. It scavenges free radicals, increases the concentration of glutathione, reduces lipid peroxidation, and thus limits oxidative stress. Thanks to these properties, quercetin may reduce the risk of neurodegenerative and cardiovascular diseases [34]. It is also known for its antibacterial, anticancer, and antiangiogenic activity [35], and it plays an important role in eliminating mycotoxins, thus protecting plant cells from damage [36]. A study by Sharma et al. [37] showed that quercetin-3-*O*-rutinoside can considerably protect the digestive tract from damage caused by gamma radiation. The authors confirmed the interaction of quercetin-3-*O*-rutinoside with essential antioxidant and anti-inflammatory proteins. The interaction of all tested antioxidant proteins (heme oxygenase-1, glutathione S-transferase, glutamate-cysteine ligase catalytic subunit, and thioredoxin reductase 1) significantly increased in the presence of quercetin-3-*O*-rutinoside [37]. Quercetin-3-*O*-rutinoside was a potent antioxidant, as it effectively quenched free radicals and efficiently chelated iron ions [38].

Anthocyanins are flavonoids commonly found in fruits and vegetables. Their presence in fruits is manifested by the red, blue, or purple color. These compounds exert strong antioxidant activity and play an important health-promoting role. They also protect the plants against abiotic and biotic stresses [39]. Anthocyanins were the least abundant polyphenolic compounds in the investigated fruits, with a maximum amount of up to 8.49%, depending on the cultivar. Their content in the investigated *P. domestica* cultivars ranged from 3.24 to 53.14 mg/100 g d.w. (Table 2). As all fruits of the *P. domestica* cultivars had light flesh, all detected anthocyanins accumulated in the fruit skin. According to Michalska et al. [40], the content of anthocyanins, responsible mainly for *P. domestica* fruit color, ranged from 18 to 170 mg/100 g d.w. The analyzed *P. domestica* samples contained four anthocyanins, with dominant cyanidin-3-*O*-rutinoside (51.03%). Their highest content was determined in cvs. Ś18, Ś30, Ś41, Ś32, and Ś16 (>20 mg/100 g d.w.), and the lowest in cvs. Ś28, Ś24, Ś42, Ś27, Ś15, Ś21, Ś23, and Ś7 (<5 mg/100 g d.w.). Another identified anthocyanin was cyanidin-3-*O*-galactoside, which accounted for 16.28% of all anthocyanins. Its highest content was found in cvs. Ś32, Ś39, and Ś31 (>9 mg/100 g d.w.), and the lowest in cvs. Ś21, Ś9, Ś15, Ś27, Ś13, Ś38, Ś22, Ś8 (<1 mg/100 g d.w.). The remaining anthocyanins accounted for 14.95% of their total pool. However, their content did not exceed 10 mg/100 g. These data are in line with the reports of Tomić et al. [18], who found cyanidin-3-*O*-glucoside and -3-*O*-rutinoside to be the two most common anthocyanins in *P. domestica* fruit.

### 3.3. Antioxidant Activity

#### 3.3.1. Antioxidant Capacity

Antioxidant activity is the ability to scavenge free radicals and prevent oxidative damage [41]. The antioxidant capacity strongly correlates with the content of phenolic compounds. The antioxidant capacity of the *P. domestica* cultivars was assessed with two independent assays, ABTS^o+^ and FRAP, with different mechanisms of action (Table 4). Total antioxidant activity in the investigated cultivars ranged from 2.20 to 7.71 mmoL Trolox/100 g and 3.25 to 13.28 mmoL Trolox/100 g for ABTS^o+^ and FRAP assays, respectively. The highest ABTS^o+^ activity was determined for cvs. Ś11, Ś2, Ś13, Ś12, Ś39, and Ś20 (>6 mmoL Trolox/100 g), and the lowest for cvs. Ś41, Ś15, Ś31, and Ś22 (<3 mmol Trolox/100 g). The highest FRAP activity was found in cvs. Ś11, Ś13, Ś2, Ś20, Ś32, Ś23, Ś12, Ś27, and Ś39 (>10 mmoL Trolox/100 g), and the lowest in cvs. Ś41, Ś31, Ś15, Ś17, and Ś10 (<6 mmoL Trolox/100 g). The cultivar with the highest ABTS^o+^ and FRAP activity was Ś11, and the lowest activity for both assays was noted in cv. Ś41 (Table 4).

According to Nowicka et al. [27], ABTS^o+^ activity in various peach cultivars ranged from 2.04 to 6.65 mmoL Trolox/100 g d.w. In cherries [42], antioxidant activity assessed with the ORAC assay reached 8.13 to 38.11 mmoL TE/100 g d.w. The ABTS^o+^ assay ranged from 3.72 to 18.40 mmoL TE/100 g d.w. For the FRAP assay, it ranged from 1.93 to 12.95 mmoL TE/100 g d.w. Fig fruits and brevas [16] yielded ORAC values between 0.46 and 1.33 mmoL Trolox/100 g d.w, while in the fruits of Japanese quince, the antioxidant activity was several times higher [43].

#### 3.3.2. Antidiabetic and Antiobesity Properties and Inhibition of Lipoxygenase

Human pancreatic α-amylase and intestinal α-glucosidase are responsible for the hydrolysis of carbohydrates into absorbable simple sugars. Inhibition of these enzymes lowers blood glucose levels by limiting the breakdown of polysaccharides into glucose [44]. IC_50_ (mg/mL) of α-amylase inhibition in the analyzed *P. domestica* fruits ranged from 2.63 (Ś34) to 61.53 (Ś41). It was not measured for cvs. Ś1, Ś2, Ś3, Ś8, Ś9, Ś11, Ś12, Ś13, Ś24, Ś28, Ś33, Ś36, and Ś39. For α-glucosidase, IC_50_ oscillated between 0.19 (Ś12) and 24.07 (Ś41), and the parameter was not assessed in cvs. Ś2, Ś6, Ś7, Ś8, Ś9, and Ś43 (Table 4). IC_50_ is the concentration of a substance at which 50% of a specific biological or biochemical function is inhibited. Therefore, the lower IC_50_, the smaller the active substance necessary to achieve the desired effect. The inhibition of α-amylase is due to the activity of bioactive plant compounds, such as polyphenolic glycosides, polysaccharides, steroids, and terpenoids [44]. α-Amylase causes postprandial hyperglycemia and increases blood glucose levels, supporting digestion by breaking down polysaccharide molecules into glucose and maltose [45]. De Sales et al. [46] showed that crude extracts and isolated compounds from plant sources could inhibit α-amylase, and flavonoids exhibited the greatest inhibition potential related to the number of hydroxyl groups in their molecules. Of the naturally occurring flavonoid compounds investigated by Kim et al. [47], the most potent inhibitors of α-amylase and α-glucosidase were luteolin, amentoflavone, luteolin 7-*O*-glucoside, and daidzein. Luteolin at 0.5 mg/mL inhibited α-glucosidase by 36%. Zhang et al. [48], who investigated different cultivars of peaches, found that the fruits inhibited α-glucosidase due to the presence of polyphenolic compounds (chlorogenic acid, neochlorogenic acid, caffeoylquinic acid, 3-*O*-feruloylquinic acid, catechin, procyanidin C1, procyanidin B1, procyanidin dimer, procyanidin trimer isomer 1, procyanidin trimer isomer 2, procyanidin B2, and prunus inhibitor b). It was also found that the type of phenolic compounds plays an important role in inhibiting α-glucosidase. Inhibition of this enzyme is one of the main strategies for countering the metabolic changes associated with hyperglycemia and type 2 diabetes. Phenolic compounds in fruits and vegetables can affect digestive enzymes involved in the hydrolysis of dietary carbohydrates. In addition, they contribute to the effective prevention of hyperglycemia by limiting lipid absorption [27]. In a study by Nowicka et al. [27], selected peach cultivars showed an inhibitory potential against α-amylase ranging from 1.41 to 4.55 mg/mL, and for α-glucosidase IC_50_ ranged from 1.31 mg/mL to 10.51 mg/mL. *P. domestica* fruits were less effective in inhibiting α-amylase and α-glucosidase. Only a few cultivars (Ś1, Ś5, Ś10) showed inhibitory activity below 0.7 mg/mL (Table 4).

Pancreatic lipase is a key enzyme responsible for the hydrolysis of dietary fats to monoacylglycerols and free fatty acids. This helps reduce overweight and obesity in patients with diabetes by significantly modulating the inhibitory effects of fat absorbed into the bloodstream [49]. In addition, the enzyme is advocated as a weight-lowering agent. *P. domestica* fruits efficiently inhibited pancreatic lipase, but this ability was cultivar-dependent (*p* < 0.05). IC_50_ [mg/mL] for pancreatic lipase ranged from 0.5 (Ś35) to 8.2 (Ś1) (Table 4). The inhibitory potential of pancreatic lipase in peaches examined by Nowicka et al. [27] was between 0.25 and 1.39 mg/mL. Turkiewicz et al. [50] reported that IC_50_ for pancreatic lipase in quince fruits ranged from 0.04 to 0.35 mg/mL, depending on the cultivar.

Lipoxygenases (LOXs) are enzymes that catalyze the oxidation of polyunsaturated fatty acids to hydroperoxides [51]. They play an important role in stimulating inflammatory reactions in the human body. Inflammation can be caused by excessive amounts of reactive oxygen species, which stimulate the release of cytokines and subsequent activation of LOX. Studies on the inhibition of lipoxygenases involved in synthesising prostaglandins and leukotrienes were conducted to identify the possibility of preventing conditions such as stroke, cancer, and cardiovascular and neurodegenerative diseases [51]. IC_50_ lipoxygenase inhibition in the tested *P. domestica* fruits ranged from 4.19 (Ś11) to 32.67 (Ś33) (Table 4)—the cvs. Ś33, Ś17, Ś8, Ś31, Ś15, and Ś37 were characterized by the highest enzyme inhibition capacity (>18.5), while the cvs. Ś11, Ś25, Ś36, Ś43, and Ś6 were the least efficient in this respect (IC_50_ < 6). Polyphenols can inhibit lipoxygenase activity by binding to the hydrophobic active site, scavenging lipid radicals, and interacting with the hydrophobic fatty acid substrate [51].

#### 3.3.3. Inhibition of AChE and BuChE Activity

A potential therapeutic strategy in Alzheimer’s and Parkinson’s diseases is to increase cholinergic levels in the brain by inhibiting the biological activity of acetylcholinesterase (AChE) and butyrylcholinesterase (BuChE). Therefore, it is important that the diet of people who suffer from these conditions contains potential inhibitors of these enzymes to increase the acetylcholine content in cholinergic synapses and improve nerve conduction [17,49]. Acetylcholinesterase, present in the neuronal synapses of the central nervous system, is a key enzyme in the cholinergic system that terminates the transmission of nerve impulses. Butyrylcholinesterase, as an enzyme associated with glial cells, endothelial cells, neurons, and senile plaques, plays a minor role in the regulation of acetylcholine levels in the brain, but its activity gradually increases in patients with Alzheimer’s disease, while the activity of AChE remains unchanged or decreases [27,52]. The source of substances inhibiting the activity of acetylcholinesterase and butyrylcholinesterase are, among others, biologically active compounds found in plants [52]. Molecular docking showed that polyphenols inhibit the activity of AChE and BuChE, offering neuroprotection and improvement of cognitive functions in Alzheimer’s and dementia [53]. For this reason, we performed additional experiments to assess *P. domestica* potential to inhibit AChE and BuChE. Inhibition of AChE, expressed as IC_50_ (mg/mL), ranged from 7.62 (Ś19) to 61.82 (Ś36), and it was not assessed in the sample Ś3 (Table 4). IC_50_ for the inhibition of BuChE ranged from 15.60 (Ś29) to 75.73 (Ś36), and the effect was not demonstrated in the Ś35 sample. These results confirmed that *P. domestica* fruits are not the most effective inhibitors of AChE or BuChE. Raw materials with strong AChE inhibition capacity of over 80% at 0.1 mg/mL include, for example, *Rhei radix et rhizome*, *Polygoni multiflori radix*, *Salviae miltiorrhiza radix*, *Radix Paeoniae alba*, *Radix Paeonie rubra*, *Chelidonii herba*, *Corydalis intermediae bulbus*, *Corydalis intermediae herba*, or *Corydalis cavae bulbus* [52]. In their study on polyphenols in *Phyllanthus emblica Linn* fruit, Wu et al. [54] reported that myricetin, quercetin, fisetin, and gallic acid were highly efficient in inhibiting AChE. Individual plant compounds were found to exert a specific therapeutic effect, which can be potentiated using the compounds in the right combinations [27]. In comparison with other fruits, that is, peaches (AChE: 4.51–42.90 and for BuChE: 8.85–18.79 mg/mL) [27] and quince (mean inhibition values for AChE 13.24, and BuChE 15.32 mg/mL) [52], we concluded that the analyzed *Prunus* fruits were less efficient at inhibiting the cholinoesterases.

### 3.4. The Elements of Primary Component Analysis

**Figure 1:**
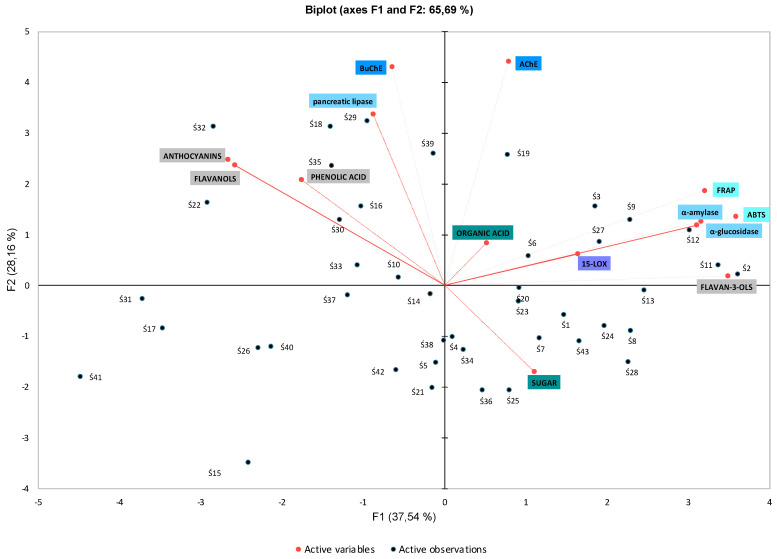
Analysis of the main components (PCA) biplot of *P. domestica* fruits and phenolic compounds, sugars, and organic acids, FRAP and ABTS^o+^ activity, antidiabetic, antiobesity and lipoxygenase inhibition, inhibition of AChE and BuChE activity. Code of the sample, see Section 2.1. Plant material.

Principal component analysis (PCA) included the following parameters: mean content of sugars, organic acids, phenolic compounds, effects of biological activity (antioxidant [ABTS^o+^, FRAP]), inhibition potential of α-amylase, α-glucosidase, pancreatic lipase, lipoxygenase, AChE, and BuChE, and the examined *P. domestica* cultivars. The PCA model (Figure 1) presents the most important variables and explains the relationships between 43 *P. domestica* cultivars, allowing for identifying group patterns. The biplot indicates that 65.69% of the total data variance is represented by F1 and F2. Of these two major components, F1 explains 37.54% of the total variance, and F2 explains 28.16%.

Cluster 1: Seven cultivars: Ś2, Ś3, Ś6, Ś9, Ś11, Ś19, and Ś27 showed a considerable potential to inhibit α-amylase, α-glucosidase, 15-LOX, and AChE, and high activity of FRAP and ABTS^o+^, which was associated with their content of flavan-3-ols and organic acids. The first principal axis showed the strongest correlations with FRAP, ABTS^o+^, flavan-3-ol levels and α-amylase and α-glucosidase inhibition levels. The Pearson test confirmed a correlation between flavan-3-ols and: FRAP and ABTS^o+^ (0.5 and 0.5, respectively), α-amylase, and α-glucosidase (0.4 and 0.4, respectively).

Cluster 2: Eight cultivars: Ś16, Ś18, Ś22, Ś29, Ś30, Ś32, Ś35, and Ś39 exhibited high content of flavonols, phenolic acids, and anthocyanins, thanks to which they showed inhibitory activity against pancreatic lipase and BuChE. Correlations were found between pancreatic lipase, BuChE activity, phenolic acid, anthocyanins, and flavonols.

Cluster 3: Twelve cultivars: Ś5, Ś14, Ś15, Ś17, Ś21, Ś26, Ś31, Ś37, Ś40, Ś41, Ś41 and Ś42, exhibited low content of flavan-3-ols and organic acids and low activity of FRAP and ABTS^o+^.

Cluster 4: Thirteen cultivars: Ś1, Ś4, Ś7, Ś8, Ś13, Ś20, Ś23, Ś24, Ś25, Ś28, Ś34, Ś36, and Ś43, were characterized by a high content of sugars (i.e., cv. Ś25 content 16.51 g/100 g total sugars), low content of anthocyanins, flavonols, and phenolic acids. Moreover, similarly to Cluster 3, they had a low inhibitory activity toward the investigated enzymes (pancreatic lipase, α-amylase, α-glucosidase, 15-LOX, AChE, and BuChE) and antioxidant activity (FRAP and ABTS^o+^). Sugar content correlated with lipoxygenase and BuChE activity. However, some cvs. (Ś13 or Ś20) still present high antioxidant potential and a relatively high content of flavan-3-ols.

PCA confirmed significant differences in the chemical composition of *P. domestica* fruit depending on the cultivar. The analysis made it possible to indicate common features of the examined cultivars and to categorize the fruits into those with a higher content of polyphenolic compounds and higher biological activity, into more sweet or sour cultivars, and into cultivars characterized by a high and low ability to inhibit α-amylase, α-glucosidase, pancreatic lipase, lipoxygenase, AChE, and BuChE.

**Figure 2:**
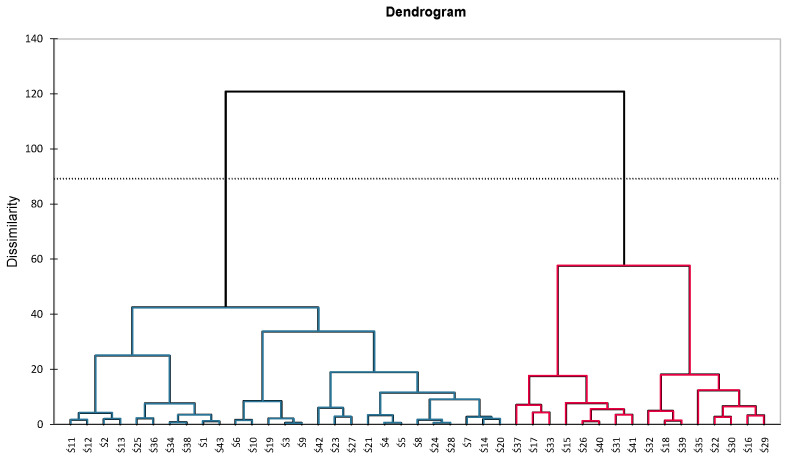
Dendrogram of agglomeration hierarchical clustering (AHC) for the fruits of selected cultivars of *P. domestica*. Code of the sample, see Section 2.1. Plant material.

We also performed agglomeration and hierarchical clustering to summarize the differences in chemical compounds and biological activity among the *P. domestica* cultivars. The AHC dendrogram is shown in Figure 2. The binary cluster tree clearly shows the differences between the cultivars. The line at 73% in the graph represents an automatic truncation, showing two homogeneous groups. The dendogram presents two groups. The first is more diverse and consists of 27 *P. domestica* cultivars. The groups are made up of cultivars that show a large diversity concerning the analyzed compounds, which confirms the relationships shown in the PCA.

## 4. Conclusions

Our study confirmed significant differences in chemical composition, phenolic compounds content, and biological properties of 43 *P. domestica* cultivars. We identified 19 phenolic compounds, including procyanidins, belonging to four groups: phenolic acids (neochlorogenic, cryptochlorogenic, chlorogenic, 3-caffeoylshikimic, and 3-feruloylquinic acid), flavonols (quercetin of -pentoside-hexoside, -3-*O*-galactoside, -3-*O*-glucoside, -3-*O*-rutinoside, -3-*O*-arabinoside, 3-*O*-rhamnoside, 3-*O*-penthoside-rhamnoside), flavan-3-ols (procyanidin B1 and B3, (+)-catechin), and anthocyanins (cyanidin-3-*O*-galactoside, -3-*O*-glucoside, -3-*O*-rutinoside, and peonidin-3-*O*-glucoside). *P. domestica* fruits were confirmed to be a rich source of phenolic compounds, particularly flavan-3-ols (16.70 to 83.79% of total phenolics content) and phenolic acids (10.47 to 69.08%). The cultivars that accumulated the greatest total amounts of biologically active compounds were Ś16, Ś17, Ś18, and Ś11. The assessment of antioxidant capacity identified cv. Ś11 had the highest ABTS^o+^ and FRAP activity, which was related to its high content of phenolic compounds, especially flavan-3-ols compounds. The analyzed cultivars more effectively inhibited AChE (IC_50_ = 7.62–61.82) than BuChE (IC_50_ = 15.60–75.73). *P. domestica* fruits are a good source of biologically active compounds and provide several health benefits, which make them a desirable element of a daily diet as fresh fruits during the season or some prepared foods such as, i.e., juices, smoothies, dried and others. The fruits of *Prunus domestica* are rich in flavan-3-ols, which contribute to their ability to inhibit α-amylase, α-glucosidase, and lipoxygenase, and rich in phenolic acid, which contributes to their ability to inhibit pancreatic lipase. For these reasons, they are helpful in the prevention of many noncommunicable diseases, particularly chronic diseases of the cardiovascular system, type 2 diabetes, gastrointestinal diseases, and some cancers.

## Figures and Tables

**Table 1:** Carbohydrates and organic acid (g/100 g d.w.) of different *Prunus domestica* cultivars.

Sample	Carbohydrates ^†^					Organic Acid ^†^						
	Fructose	Sorbitol	Glucose	Saccharose	Total	Oxalic Acid	Citric Acid	Malic Acid	Quinic Acid	Succinic Acid	Formic Acid	Total
Ś1	3.52 ± 0.35 g–j	0.69 ± 0.11 g–j	2.94 ± 0.12 f–l	1.84 ± 0.23 n–r	8.98 i–l	0.15 ± 0.02 k–n	nd f	1.24 ± 0.21 g–l	0.43 ± 0.10 m–p	0.09 ± 0.03 ef	0.06 ± 0.01 d	1.97 h–o
Ś2	2.72 ± 0.54 k–p	nd r	3.85 ± 0.21 b–d	2.23 ± 0.18 l–p	8.80 i–m	0.17 ± 0.02 i–l	0.07 ± 0.01 a	1.01 ± 0.28 l–q	0.42 ± 0.11 m–p	0.07 ± 0.02 gh	0.04 ± 0.01 f	1.80 j–p
Ś3	3.21 ± 0.29 h–m	1.04 ± 0.10 cd	3.79 ± 0.15 b–d	3.25 ± 0.32 k	11.28 e–h	0.32 ± 0.06 ef	0.01 ± 0.00 e	1.25 ± 0.11 g–l	0.73 ± 0.01 g–j	0.09 ± 0.03 ef	0.08 ± 0.01 b	2.49 b–f
Ś4	2.05 ± 0.32 p–s	0.60 ± 0.09 j–m	5.87 ± 0.14 a	3.09 ± 0.33 kl	11.61 d–h	0.33 ± 0.04 e	0.02 ± 0.00 d	0.87 ± 0.21 p–s	1.31 ± 0.26 cd	0.09 ± 0.03 ef	0.13 ± 0.03 a	2.76 bc
Ś5	2.51 ± 0.10 m–r	0.74 ± 0.04 g–i	4.35 ± 0.26 b	2.55 ± 0.12 k–o	10.15 g–k	0.16 ± 0.02 j–m	0.01 ± 0.00 e	0.76 ± 0.09 r–u	0.61 ± 0.09 i–k	0.05 ± 0.01 ij	0.02 ± 0.00 h	1.62 m–q
Ś6	2.06 ± 0.48 p–s	1.61 ± 0.06 a	3.48 ± 0.32 c–f	5.31 ± 0.15 ij	12.46 c–f	0.28 ± 0.11 fg	0.01 ± 0.00 e	1.34 ± 0.19 e–j	0.54 ± 0.11 k–n	0.07 ± 0.02 gh	nd j	2.24 d–j
Ś7	2.00 ± 0.49 p–s	0.51 ± 0.08 l–n	3.78 ± 0.54 b–e	5.71 ± 0.67 h–j	12.00 c–g	0.21 ± 0.06 hi	0.02 ± 0.00 d	1.02 ± 0.11 l–q	1.22 ± 0.11 d	0.06 ± 0.01 hi	0.07 ± 0.03 c	2.61 b–d
Ś8	2.04 ± 0.19 p–s	0.03 ± 0.00 qr	3.21 ± 0.19 e–i	5.32 ± 0.48 ij	10.60 e–i	0.28 ± 0.01 fg	0.01 ± 0.00 e	1.15 ± 0.19 i–o	0.96 ± 0.16 e	0.06 ± 0.03 hi	0.08 ± 0.03 b	2.56 b–e
Ś9	4.06 ± 0.20 fg	nd r	1.81 ± 0.47 q–u	5.89 ± 0.59 g–i	11.76 d–h	0.06 ± 0.11 rs	0.01 ± 0.00 e	1.52 ± 0.23 c–f	0.77 ± 0.06 f–h	0.07 ± 0.04 gh	0.04 ± 0.01 f	2.47 b–g
Ś10	5.32 ± 0.48 cd	nd r	0.99 ± 0.11 w	6.38 ± 0.72 e–h	12.69 b–e	0.09 ± 0.01 p–s	0.01± 0.00 e	1.19 ± 0.16 i–m	0.20 ± 0.02 t	0.07 ± 0.05 gh	0.01 ± 0.00 i	1.57 o–q
Ś11	2.88 ± 0.29 j–o	nd r	2.98 ± 0.16 f–k	8.75 ± 0.39 a	14.61 ab	0.35 ± 0.11 e	0.02 ± 0.00 d	1.23 ± 0.31 h–l	0.80 ± 0.01 fg	0.06 ± 0.01 hi	0.04 ± 0.01 f	2.50 b–f
Ś12	1.79 ± 0.34 rs	nd r	1.92 ± 0.16 p–u	8.46 ± 0.54 a	12.17 c–g	0.10 ± 0.02 o–r	nd f	0.92 ± 0.21 n–r	0.21 ± 0.04 st	0.09 ± 0.03 ef	0.02 ± 0.00 h	1.34 q
Ś13	3.09 ± 0.24 h–n	1.68 ± 0.02 a	6.19 ± 0.54 a	1.41 ± 0.28 p–s	12.38 c–f	0.25 ± 0.04 gh	0.05 ± 0.01 b	1.52 ± 0.15 c–f	0.76 ± 0.16 f–i	0.08 ± 0.01 fg	0.08 ± 0.03 b	2.74 bc
Ś14	1.97 ± 0.38 q–s	nd r	1.58 ± 0.21 t–v	7.54 ± 0.19 b–d	11.09 e–h	0.11 ± 0.05 n–q	0.02 ± 0.00 d	1.19 ± 0.32 i–m	0.25 ± 0.05 r–t	0.07 ± 0.01 gh	0.03 ± 0.00 g	1.68 k–q
Ś15	3.25 ± 0.18 h–l	nd r	1.14 ± 0.21 vw	2.96 ± 0.32 kl	7.35 l–p	0.36 ± 0.02 e	0.04 ± 0.00 c	0.61 ± 0.16 tu	0.44 ± 0.19 l–o	0.09 ± 0.03 ef	0.04 ± 0.01 f	1.60 n–q
Ś16	3.26 ± 0.43 h–l	0.87 ± 0.03 ef	3.09 ± 0.12 f–j	1.73 ± 0.22 o–r	8.96 i–l	0.25 ± 0.02 gh	0.01 ± 0.00 e	1.32 ± 0.10 f–k	0.41 ± 0.12 n–q	0.07 ± 0.01 gh	0.02 ± 0.00 h	2.09 f–l
Ś17	1.50 ± 0.27 s	0.47 ± 0.7 n	1.89 ± 0.17 p–u	1.85 ± 0.28 n–r	5.71 p	0.18 ± 0.01 i–l	0.01 ± 0.00 e	1.20 ± 0.25 i–l	0.55 ± 0.11 k–n	0.11 ± 0.01 cd	0.08 ± 0.03 b	2.15 e–j
Ś18	3.19 ± 0.29 h–m	0.93 ± 0.11 de	2.31 ± 0.12 m–r	2.28 ± 0.32 l–p	8.70 i–n	0.49 ± 0.02 c	0.01 ± 0.00 e	1.76 ± 0.21 bc	0.55 ± 0.01 k–n	0.09 ± 0.02 ef	nd j	2.90 b
Ś19	3.71 ± 0.19 f–i	0.80 ± 0.37 fg	2.97 ± 0.32 f–k	2.21 ± 0.37 l–p	9.70 h–k	0.33 ± 0.03 e	0.01 ± 0.00 e	1.51 ± 0.22 d–f	0.49 ± 0.15 k–o	0.09 ± 0.02 ef	0.02 ± 0.00 h	2.45 b–g
Ś20	2.88 ± 0.39 j–o	0.46 ± 0.29 no	2.38 ± 0.18 l–q	4.83 ± 0.47 j	10.54 f–i	0.15 ± 0.10 k–n	nd f	0.74 ± 0.17 r–u	1.00 ± 0.17 e	0.04 ± 0.01 j	0.02 ± 0.00 h	1.96 h–o
Ś21	2.96 ± 0.25 j–n	0.49 ± 0.05 mn	2.75 ± 0.19 h–m	1.25 ± 0.38 q–s	7.44 l–p	0.12 ± 0.13 m–p	0.01 ± 0.00 e	1.61 ± 0.21 cd	0.28 ± 0.07 p–t	0.10 ± 0.03 de	0.02 ± 0.00 h	2.14 e–j
Ś22	4.30 ± 0.50 ef	0.34 ± 0.01 o	1.98 ± 0.21 p–u	1.54 ± 0.28 p–r	8.16 k–o	0.07 ± 0.11 q–s	0.01 ± 0.00 e	1.48 ± 0.15 d–g	0.64 ± 0.16 h–k	0.11 ± 0.03 cd	0.02 ± 0.00 h	2.33 c–h
Ś23	3.55 ± 0.19 g–j	0.14 ± 0.02 pq	3.29 ± 0.34 d–h	2.84 ± 0.48 k–m	9.82 h–k	0.60 ± 0.23 a	0.01 ± 0.00 e	1.96 ± 0.18 ab	1.44 ± 0.21 c	0.07 ± 0.01 gh	0.04 ± 0.00 f	4.12 a
Ś24	3.02 ± 0.39 i–n	0.52 ± 0.11 l–n	1.78 ± 0.11 r–u	2.99 ± 0.27 kl	8.32 j–n	0.42 ± 0.05 d	0.01 ± 0.00 e	1.34 ± 0.32 e–j	0.57 ± 0.13 k–m	0.11 ± 0.01 cd	0.07 ± 0.03 c	2.52 b–f
Ś25	5.30 ± 0.79 cd	0.46 ± 0.05 no	3.87 ± 0.32 bc	6.89 ± 0.33 d–f	16.51 a	0.14 ± 0.07 l–o	0.01 ± 0.00 e	0.79 ± 0.17 q–t	0.37 ± 0.09 o–r	0.05 ± 0.03 ij	0.01 ± 0.00 i	1.38 pq
Ś26	3.32 ± 0.55 h–k	1.15 ± 0.11 bc	2.93 ± 0.21 f–l	7.24 ± 0.52 c–e	14.64 ab	0.09 ± 0.02 p–s	0.01 ± 0.00 e	1.09 ± 0.21 k–p	0.36 ± 0.08 o–s	0.06 ± 0.03 hi	0.04 ± 0.00 f	1.66 l–q
Ś27	2.21 ± 0.41 o–s	0.18 ± 0.03 p	1.11 ± 0.22 vw	2.64 ± 0.57 k–n	6.14 op	0.33 ± 0.03 e	0.02 ± 0.00 d	1.86 ± 0.11 ab	1.82 ± 0.11 b	0.12 ± 0.03 c	0.04 ± 0.00 f	4.19 a
Ś28	6.27 ± 0.26 b	0.56 ± 0.02 k–n	2.85 ± 0.31 g–m	0.12 ± 0.02 u	9.80 h–k	0.12 ± 0.01 m–p	0.02 ± 0.00 d	0.91 ± 0.32 o–r	0.36 ± 0.02 o–s	0.05 ± 0.01 ij	0.07 ± 0.08 c	1.55 o–q
Ś29	3.21 ± 0.12 h–m	0.18 ± 0.11 p	1.44 ± 0.28 u–w	2.03 ± 0.26 m–q	6.85 m–p	0.10 ± 0.01 o–r	0.02 ± 0.00 d	1.37 ± 0.12 d–j	0.26 ± 0.03 q–t	0.04 ± 0.01 j	0.03 ± 0.00 g	1.83 i–p
Ś30	4.80 ± 0.32 de	nd r	1.73 ± 0.43 s–u	8.14 ± 0.33 a–c	14.66 ab	0.19 ± 0.02 i–k	0.02 ± 0.00 d	1.57 ± 0.25 c–e	0.25 ± 0.01 r–t	0.10 ± 0.01 de	0.01 ± 0.00 i	2.14 e-j
Ś31	7.00 ± 0.12 a	0.09 ± 0.11 p–r	3.14 ± 0.66 f–j	0.03 ± 0.00 u	10.27 g–j	0.10 ± 0.01 o–r	0.02 ± 0.00 d	0.83 ± 0.11 q–t	0.45 ± 0.08 l–o	0.05 ± 0.01 ij	0.02 ± 0.00 h	1.48 pq
Ś32	2.44 ± 0.38 n–r	nd r	1.43 ± 0.28 u–w	1.62 ± 0.02 p–r	5.49 p	0.08 ± 0.02 p–s	0.01 ± 0.00 e	0.95 ± 0.10 m–r	0.90 ± 0.09 ef	0.10 ± 0.03 de	0.03 ± 0.00 g	2.07 f–m
Ś33	5.66 ± 0.63 bc	0.77 ± 0.11 f–h	3.09 ± 0.10 f–j	0.15 ± 0.01 tu	9.67 h–k	0.25 ± 0.05 gh	0.01 ± 0.00 e	1.16 ± 0.25 i–n	0.75 ± 0.10 f–i	0.11 ± 0.03 cd	0.07 ± 0.03 c	2.37 c–h
Ś34	7.55 ± 0.38 a	nd r	3.43 ± 0.29 c–f	1.11 ± 0.11 rs	12.09 c–g	0.05 ± 0.01 s	0.01 ± 0.00 e	0.54 ± 0.03 u	0.63 ± 0.09 h–k	0.12 ± 0.01 c	0.04 ± 0.00 f	1.41 pq
Ś35	2.57 ± 0.28 l–q	0.67 ± 0.14 h–k	2.06 ± 0.21 o–t	0.59 ± 0.02 s–u	5.90 p	0.11 ± 0.01 n–q	0.01 ± 0.00 e	2.09 ± 0.27 a	0.26 ± 0.08 q–t	0.07 ± 0.02 gh	0.07 ± 0.01 c	2.62 b–d
Ś36	2.42 ± 0.72 n–r	0.76 ± 0.09 f–h	1.91 ± 0.21 p–u	7.40 ± 0.43 cd	12.49 c–f	0.14 ± 0.02 l–o	0.01 ± 0.00 e	0.64 ± 0.04 s–u	0.75 ± 0.17 f–i	0.06 ± 0.03 hi	0.05 ± 0.01 e	1.66 l–q
Ś37	2.56 ± 0.28 l–q	nd r	2.17 ± 0.32 n–s	3.33 ± 0.16 k	8.06 k–o	0.11 ± 0.03 n–q	0.02 ± 0.00 d	0.81 ± 0.03 q–t	0.59 ± 0.08 j–l	0.07 ± 0.02 gh	0.05 ± 0.01 e	1.66 l–q
Ś38	3.36 ± 0.32 g–k	1.24 ± 0.23 b	2.71 ± 0.25 i–n	6.69 ± 0.32 d–g	14.00 bc	0.17 ± 0.06 i–l	0.01 ± 0.00 e	1.25 ± 0.11 g–l	0.43 ± 0.08 m–p	0.11 ± 0.01 cd	0.04 ± 0.01 f	2.03 g–n
Ś39	3.30 ± 0.11 h–k	1.05 ± 0.11 cd	2.41 ± 0.21 k–p	1.40 ± 0.37 p–s	8.16 k–o	0.16 ± 0.02 j–m	0.01 ± 0.00 e	1.09 ± 0.10 k–p	1.04 ±0.12 e	0.09 ± 0.01 ef	0.02 ± 0.00 h	2.41 c–h
Ś40	5.19 ± 0.51 cd	nd r	0.06 ± 0.00 x	8.40 ± 0.65 ab	13.65 b–d	0.19± 0.03 i–k	0.01 ± 0.00 e	1.39 ± 0.16 d–i	0.40 ± 0.11 n–r	0.07 ± 0.03 gh	0.05 ± 0.01 e	2.13 e–k
Ś41	3.30 ± 0.37 h–k	0.15 ± 0.03 pq	2.58 ± 0.21 j–o	6.05 ± 0.87 f–i	12.08 c–g	0.20 ± 0.02 ij	0.02 ± 0.00 d	1.24 ± 0.11 g–l	0.99 ± 0.03 e	0.14 ± 0.01 b	0.05 ± 0.01 e	2.63 b–d
Ś42	2.56 ± 0.09 l–q	0.62 ± 0.02 i–l	2.42 ± 0.17 k–p	1.04 ± 0.25 r–t	6.63 n–p	0.41 ± 0.03 d	nd f	1.45 ± 0.18 d–h	2.22 ± 0.32 a	0.18 ± 0.03 a	0.04 ± 0.00 f	4.33 a
Ś43	3.80 ± 0.27 f–h	0.71 ± 0.10 g–j	3.38 ± 0.27 c–g	5.69 ± 0.33 h–j	13.59 b–d	0.55 ± 0.00 b	nd f	1.14 ± 0.11 j–o	0.44 ± 0.11 l–o	0.10 ± 0.03 de	0.04 ± 0.00 f	2.28 d–i

^†^ means value of n = 3 independent repetition; nd—not detected; a, b, c,… values followed by the same letter within a column are not significantly different (*p* < 0.05; Tukey’s test).

**Table 2:** Properties of individual main phenolic compounds of the *Prunus domestica* fruits using their spectral characteristics by LC-MS Qtof in the negative characterization of flavan-3-ols (280 nm), phenolic acid (320 nm), flavonols (360 nm) and positive characterization of anthocyanins (520 nm) ionization mode.

t_R_ (min)	Assigned Identity	Molecular Ion [M-H]^−^ (*m*/*z*)	Main Ions MS/MS (*m*/*z*)
Flavan-3-ols			
2.30	(+)-Catechin ^†^	289.0143	245.0367
3.30	Procyanidin dimer (B1) ^†^	577.0538	289.0143
4.50	Procyanidin dimer (B3) ^†^	577.0311	289.0152
Phenolic acids			
3.71	Neochlorogenic acid ^†^	353.0532	191.0232
3.92	Cryptochlorogenic acid ^†^	353.0534	191.0241
4.02	Chlorogenic acid ^†^	353.0531	191.0254
4.83	3-Caffeoylshikimic acid	335.0713	191.0511
5.02	3-Feruloylquinic acid	367.2312	193.2101/191.2302
Flavonols			
3.36	Quercetin-3-*O*-pentoside-hexoside	595.0411	449.0311/301.0062
3.60	Quercetin-3-*O*-glucoside ^†^	463.0523	301.0062
4.20	Quercetin-3-*O*-arabinoside ^†^	433.0302	301.0666
6.62	Quercetin-3-*O*-rutinoside ^†^	609.1018	301.0133
6.78	Quercetin-3-*O*-galactoside ^†^	463.0523	301.0054
7.32	Quercetin-3-*O*-rhamnoside ^†^	447.0902	301.0054
10.22	Quercetin-*O*-pentoside-rhamnoside	579.0332	301.1803
Anthocyanins			
4.59	Cyanidin-3-*O*-galactoside ^†^	449.0324	287.0180
6.29	Cyanidin-3-*O*-glucoside ^†^	449.0646	299.0216/287.0180
6.55	Cyanidin-3-*O*-rutinoside ^†^	595.1507	287.0536
6.72	Delphinidin-3-*O*-glucoside ^†^	465.0243	303.0098
6.86	Peonidin-3-*O*-glucoside ^†^	463.1201	301.0721

^†^ t_R_, MS and MS/MS, compared with a standard compound; t_R_, retention time.

**Table 3:** Phenolic compounds (mg/100 g dw) of different *Prunus domestica* cultivars.

Sample	Flavan-3-Ols ^†^							Phenolic Acid ^†^							
	Procyanidin B1	Procyanidin B3	(+)-Catechin	PP	Other		Total	Neochlorogenic Acid	Cryptochlorogenic Acid	Chlorogenic Acid		3-Caffeoylshikimic Acid	3-Feruloylquinic Acid	Other	Total
Ś1	11.59 ± 1.11 n–p	4.15 ± 0.35 j–l	4.25 ± 0.23 kl	529.3 ± 12.3 f–h	31.55 ± 4.21 e		580.89 g–k	269.11 ± 1.32 h–k	9.45 ± 0.32 l–o	33.32 ± 1.32 i–l		5.95 ± 0.32 ef	10.47 ± 0.32 d–f	13.47 ± 0.43 j–m	341.77 i–l
Ś2	24.84 ± 2.11 ef	9.77 ± 0.34 d	nd u	1022.7 ± 24.5 a	57.15 ± 3.65 b		1114.47 a	219.20 ± 11.23 j–n	10.08 ± 0.12 k–n	24.48 ± 2.13 l–p		2.90 ± 0.23 n–s	4.92 ± 0.14 mn	2.74 ± 0.53 t	264.32 n–r
Ś3	8.88 ± 1.76 p–s	2.54 ± 0.62 o–r	2.56 ± 0.38 n–s	618.2 ± 23.1 d–f	52.43 ± 3.87 b		684.68 d–h	216.32 ± 2.64 k–n	10.41± 0.32 k–n	17.85 ± 1.56 p–t		3.77 ± 0.54 k–n	6.71 ± 0.43 i–l	17.30 ± 0.41 g–j	272.34 l–r
Ś4	19.01 ± 1.87 h–j	5.17 ± 0.34 h–j	7.57 ± 0.25 de	353.3 ± 17.5 kl	40.66 ± 2.54 cd		425.77 k–o	357.06 ± 4.44 fg	nd t	35.73 ± 2.11 h–k		4.86 ± 0.54 g–i	1.24 ± 0.32 s–u	19.34 ± 0.67 f–h	418.23 f–h
Ś5	11.03 ±1.63 o–q	2.51 ± 0.12 o–r	3.23 ± 0.23 m–o	283.7 ± 12.3 lm	10.82 ± 3.28 p–t		311.35 n–r	275.01 ± 3.32 h–j	7.94 ± 1.03 n–q	40.10 ± 1.65 g–i		3.58 ± 0.34 l–o	1.11 ± 0.11 tu	12.41 ± 0.29 k–n	340.16 i–m
Ś6	15.59 ± 1.66 j–l	3.81 ± 0.13 k–n	5.60 ± 0.45 h–j	490.2 ± 17.3 g–i	37.44 ± 4.29 d		552.70 g–l	301.18 ± 2.22 g–i	7.22 ± 0.99 n–r	24.23 ±2.43 m–q		1.63 ± 0.12 u	2.50 ± 0.24 o–t	11.85 ± 0.76 k–o	348.60 h–k
Ś7	24.79 ± 2.11 ef	6.21 ± 0.47 f–h	9.35 ± 0.11 c	238.9 ± 11.1 m–p	13.48 ± 1.32 n–s		292.80 o–s	52.29 ± 2.43 st	4.02 ± 0.65 rs	35.41 ± 1.44 h–k		2.54 ± 0.32 q–t	9.91 ± 0.15 d–f	12.03 ± 0.49 k–o	116.20 wx
Ś8	9.89 ± 1.04 o–s	3.31 ± 0.23 k–p	4.26 ± 0.15 kl	540.0 ± 13.2 f–h	4.24 ± 1.32 u–w		561.74 g–l	188.29 ± 3.22 m–p	4.02 ± 1.43 rs	15.08 ± 2.12 r–v		1.76 ± 0.11 tu	3.04 ± 0.17 o–r	5.10 ± 0.65 r–t	217.30 q–t
Ś9	23.17 ± 1.00 fg	6.15 ± 0.47 f–h	7.17 ± 0.48 e–g	741.3 ± 10.0 c	20.06 ± 2.76 h–l		797.94 cd	360.55 ± 4.23 ef	21.73 ± 0.32 e	30.57 ± 1.87 j–n		4.74 ± 0.43 h–j	5.41 ± 0.42 lm	21.98 ± 0.89 f	444.97 e–g
Ś10	15.37 ± 1.62 j–m	1.58 ± 0.21 r–t	2.88 ± 0.12 m–r	380.2 ± 13.7 jk	nd w		400.12 l–p	308.43 ± 1.43 f–h	16.33 ± 1.32 f–h	28.07 ± 1.84 j–o		3.46 ± 0.34 l–p	9.05 ± 0.18 fg	18.19 ± 0.77 f–i	383.53 g–j
Ś11	11.65 ± 1.01 m–p	4.41 ± 0.43 i–k	5.36 ± 0.43 ij	686.6 ± 12.2 cd	70.17 ± 4.10 a		778.27 c–f	463.37 ± 3.12 bc	33.83 ± 0.87 bc	34.56 ± 1.99 i–k		2.53 ± 0.24 q–u	6.01 ± 0.61 k–m	17.89 ± 0.99 f–i	558.19 cd
Ś12	48.75 ± 0.23 a	11.51 ± 0.76 c	11.36 ± 0.65 a	857.0 ± 10.4 b	52.55 ± 5.10 b		981.24 ab	92.73 ± 1.44 r–t	4.08 ± 0.54 rs	19.62 ± 1.63 o–s		2.61 ± 0.43 p–t	3.61 ± 0.36 n–p	3.10 ± 0.38 t	125.74 v–x
Ś13	16.32 ± 1.11 j–l	2.62 ± 0.43 o–r	3.55 ± 0.34 l–n	494.1 ± 10.3 g–i	17.70 ± 2.10 i–n		534.35 h–l	266.54 ± 2.54 h–k	11.36 ± 1.32 j–m	26.90 ± 1.54 k–o		3.01 ± 0.37 m–r	3.71 ± 0.28 no	10.98 ± 0.56 l–p	322.50 j–m
Ś14	23.16 ± 0.54 fg	15.86 ± 0.32 b	nd u	429.7 ± 9.9 i–k	15.58 ± 2.31 k–q		484.40 j–m	171.97 ± 2.11 n–p	5.51 ± 0.55 qr	45.83 ± 1.11 fg		2.74 ± 0.12 o–s	10.71 ± 0.37 de	9.29 ± 0.47 m–r	246.04 p–t
Ś15	4.41 ± 0.32 tu	1.77 ± 0.43 q–t	1.86 ± 0.11 st	202.3 ± 10.7 m–q	22.78 ± 2.54 g–i		233.18 p–t	237.00 ± 0.32 j–m	9.01 ± 0.99 l–p	22.38 ± 1.76 n–r		4.13 ± 0.46 i–l	2.18 ± 0.19 p–t	10.41 ± 0.38 m–q	285.11 k–q
Ś16	11.39 ± 0.43 n–p	2.78 ± 0.25 n–q	2.66 ± 0.23 n–s	394.9 ± 11.4 jk	5.88 ± 3.29 t–v		417.66 k–o	502.25 ± 1.54 b	36.21 ± 1.11 b	167.71 ± 2.54 a		16.37 ± 1.01 a	20.85 ± 0.57 a	61.38 ± 0.88 a	804.77 a
Ś17	15.51 ±0.26 j–l	2.32 ± 0.52 o–s	2.02 ± 0.15 q–s	234.2 ± 14.3 m–p	3.12 ± 0.54 vw		257.25 o–t	572.68 ± 2.15 a	54.55 ± 3.22 a	50.93 ± 1.02 ef		6.66 ± 0.22 de	3.15 ± 0.43 o–q	40.23 ± 0.46 d	728.20 b
Ś18	23.61 ± 0.54 e–g	19.42 ± 0.51 a	nd u	269.2 ± 10.5 l–n	10.60 ± 1.11 q–t		322.85 m–r	462.49 ± 2.43 bc	17.41 ± 1.05 fg	64.27 ± 1.32 cd		7.68 ± 0.54. bc	8.25 ± 0.12 gh	44.96 ± 0.29 c	605.07 c
Ś19	16.23 ± 0.21 j–l	3.43 ± 0.17 k–o	3.05 ± 0.31 m–p	513.1 ± 21.0 g–i	20.61 ± 2.73 h–k		556.50 g–l	435.02 ± 0.33 c	32.84 ± 0.76 c	46.41± 1.72 fg		3.74 ± 0.28 k–n	14.54 ± 0.32 c	36.36 ± 1.43 d	568.91 cd
Ś20	42.65 ± 0.32 b	8.28 ± 0.51 e	10.27 ± 0.56 bc	229.0 ± 10.0 m–p	15.31 ± 0.18 l–r		305.56 n–r	48.17 ± 2.65 t	nd t	23.49 ± 1.99 m–r		nd v	7.40 ± 0.37 h–k	4.25 ± 0.12 st	83.31 x
Ś21	10.89 ± 0.11 o–q	nd u	nd u	885.1 ± 46.1 b	13.96 ± 2.01 m–s		910.04 bc	157.66 ± 1.44 o–q	8.24 ± 0.22 m–q	8.81 ± 1.73 u–w		2.09 ± 0.34 s–u	3.29 ± 0.29 op	7.46 ± 0.54 p–s	187.54 s–w
Ś22	14.91 ± 0.42 k–n	nd u	1.94 ± 0.13 rs	83.5 ± 8.3 s	21.05 ± 2.82 h–j		121.45 st	423.93 ± 0.65 cd	14.34 ± 2.11 g–j	28.99 ± 1.87 j–n		5.23 ± 0.23 f–h	11.30 ± 1.11 d	20.74 ± 0.45 fg	504.53 de
Ś23	27.28 ± 0.12 de	nd u	nd u	267.4 ± 12.0 l–o	12.93 ± 1.44 n–s		307.62 n–r	193.47 ± 0.99 l–p	5.77 ± 0.32 qr	24.59 ± 0.81 l–p		nd v	3.23 ± 0.43 op	7.78 ± 0.48 o–s	234.84 p–t
Ś24	10.26 ± 0.74 o–r	3.01 ± 0.43 l–p	3.32 ± 0.25 l–n	641.9 ± 10.1 de	4.46 ± 0.54 u–w		662.98 d–i	219.18 ± 0.65 j–n	13.81 ± 0.55 h–j	15.58 ± 0.71 q–u		3.13 ± 0.29 m–r	2.68 ± 0.32 o–s	11.68 ± 0.99 k–p	266.07 m–r
Ś25	15.94 ± 0.12 j–l	nd u	4.82 ± 0.11 jk	458.8 ± 10.4 h–j	12.42 ± 1.11 o–s		491.99 i–m	105.22 ± 0.56 q–t	5.30 ± 0.99 qr	59.55 ± 0.72 de		nd v	9.77 ± 0.88 ef	19.91± 0.94 f–h	199.75 r–v
Ś26	10.76 ± 0.54 o–q	nd u	1.81 ± 0.43 st	185.2 ± 11.1 n–r	14.29 ± 0.88 m–s		212.16 q–t	192.49 ± 1.32 l–p	5.27 ± 0.54 qr	43.59 ± 0.99 f–h		2.29 ± 0.54 r–u	6.83 ± 0.27 h–l	8.18 ± 0.48 n–s	258.64 o–s
Ś27	29.74 ± 0.62 cd	6.60 ± 0.23 fg	8.22 ± 0.56 d	564.3 ± 20.1 e–g	13.13 ± 3.11 n–s		622.06 e–j	231.54 ± 2.43 j–m	nd t	nd w		3.56 ± 0.29 l–o	1.63 ± 0.29 r–t	17.14 ± 0.77 g–j	253.86 o–t
Ś28	7.57 ± 1.12 q–t	4.06 ± 0.43 j–m	3.02 ± 0.54 m–q	755.0 ± 20.5 c	21.19 ± 1.11 h–j		790.90 c–e	107.59 ± 1.99 q–s	5.80 ± 0.23 p–r	6.50 ± 0.72 vw		0.70 ± 0.54 v	3.08 ± 0.11 o–r	6.34 ± 0.56 q–t	130.02 u–x
Ś29	13.37 ± 0.48 l–o	5.40 ± 0.26 hi	6.28 ± 0.12 g–i	433.1 ± 16.4 i–k	13.26 ± 1.43 n–s		471.41 j–n	426.42 ± 1.56 c	13.73 ± 0.45 h–j	32.08 ± 1.21 i–m		6.89 ± 0.29 cd	8.20 ± 0.99 g–i	15.97± 0.77 h–k	503.29 de
Ś30	13.07 ± 0.12 l–o	1.13 ± 0.11 tu	2.27 ± 0.13 o–s	209.3 ± 10.3 m–q	3.59 ± 1.03 vw		229.36 p–t	247.05 ± 1.99 i–l	17.32 ± 0.76 fg	12.99 ± 0.63 s–v		3.91 ± 0.10 j–m	6.28 ± 1.11 j–m	10.41 ± 0.68 m–q	297.95 k–p
Ś31	1.16 ± 0.15 u	nd u	3.76 ± 0.43 lm	235.0 ± 10.7 m–p	43.40 ± 2.67 c		283.37 o–t	366.51 ± 2.54 d–f	10.26 ± 0.34 k–n	61.35 ± 0.71 cd		8.57 ± 0.10 b	7.45 ± 0.73 h–k	27.16 ± 0.99 e	481.29 ef
Ś32	10.09 ± 0.35 o–r	nd u	nd u	175.0 ± 10.3 o–s	16.77 ± 2.68 j–o		201.88 q–t	311.67 ± 2.14 f–h	14.67 ± 0.99 g–i	62.03 ± 0.29 cd		2.39 ± 0.47 r–u	5.96 ± 0.34 k–m	20.52 ± 0.68 fg	417.24 f–h
Ś33	22.93 ± 0.37 fg	7.06 0.23 f	7.21 ± 0.36 d–g	503.6 ± 11.1 g–i	25.07 ± 1.88 f–h		565.91 g–l	347.88 ± 4.21 fg	12.21 ± 0.23 i–l	68.69 ± 1.43 c		3.30 ± 0.12 l–q	16.13 ± 0.53 b	11.41 ± 0.59 l–p	459.62 ef
Ś34	8.35 ± 0.39 p–s	2.94 ± 0.43 m–q	3.09 ± 0.12 m–p	568.6 ± 10.4 e–g	28.32 ± 2.01 ef		611.35 f–j	212.83 ± 2.43 k–o	5.68 ± 0.37 qr	33.86 ± 1.72 i–k		5.66 ± 0.15 fg	4.88 ± 0.16 mn	21.07 ± 0.47 fg	283.97 k–q
Ś35	6.79 ± 0.66 r–t	nd u	0.89 ± 0.32 tu	105.6 ± 11.0 rs	4.00 ± 0.36 vw		117.35 t	137.42 ± 1.99 p–r	4.48 ± 0.29 rs	23.36 ± 1.54 m–r		2.49 ± 0.21 q–u	2.84 ± 0.43 o–r	11.80 ± 1.02 k–o	182.39 t–w
Ś36	18.86 ± 0.10 ij	6.97 ± 0.43 f	6.51 ±0.23 f–h	221.1 ± 15.3 m–p	10.34 ± 1.01 r–t		263.87 o–t	162.35 ± 1.45 n–q	6.31 ± 0.32 o–r	11.82 ± 1.24 s–v		1.78 ± 0.45 tu	4.85 ± 0.54 mn	17.10 ± 2.01 g–j	204.20 r–u
Ś37	22.75 ± 1.04 f–h	6.34 ± 0.46 f–h	5.01 ± 0.43 jk	211.0 ± 28 m–q	27.74 ± 2.01 e–g		272.85 o–t	150.02 ± 2.11 p–r	8.02 ± 0.58 n–q	82.54 ± 1.14 b		nd v	13.73 ± 1.11 c	49.54 ± 0.12 b	303.85 k–p
Ś38	31.11 ± 0.19 c	9.36 ± 0.54 de	11.18 ± 0.99 ab	274.2 ± 35 l–n	17.05 ± 2.07 j–o		342.98 m–q	362.40 ± 1.54 ef	26.86 ± 0.99 d	36.36 ± 1.63 h–j		3.63 ± 0.52 l–o	5.86 ± 1.88 lm	20.49 ± 0.43 fg	455.61 e–g
Ś39	20.90 ± 1.00 g–i	5.22 ± 0.65 h–j	7.51 ± 0.43 d–f	427.6 ± 19 i–k	15.84 ± 1.54 k–p		477.12 j–n	264.32 ± 1.78 h–k	12.74 ± 0.79 i–k	56.10 ± 1.71 de		2.80 ± 0.32 o–s	6.77 ± 0.75 h–l	14.91 ± 0.19 j–l	357.63 h–k
Ś40	10.95 ± 0.28 o–q	1.21 ± 0.11 st	2.17 ± 0.28 p–s	154.2 ± 10.2 p–s	30.93 ± 0.32 e		199.51 q–t	417.24 ± 2.66 c–e	15.40 ± 0.39 f–i	40.20 ± 1.92 g–i		4.90 ± 0.54 g–i	1.70 ± 0.12 q–t	39.17 ± 1.01 d	518.61 de
Ś41	11.27 ± 0.32 n–q	2.17 ± 0.12 p–t	3.45 ± 0.47 l–n	127.9 ± 10.3 q–s	18.69 ± 1.15 i–m		163.49 r–t	340.10 ± 3.05 fg	18.07 ± 1.07 f	30.57 ± 1.44 j–n		3.86 ± 0.73 j–m	nd u	20.81± 1.44 fg	413.41 f–i
Ś42	6.15 ± 0.11 st	1.16 ± 0.09 s–u	2.01 ± 0.18 q–s	215.6 ± 11.0 m–q	9.24 ± 1.05 s–u		134.26 p–t	63.88 ± 2.43 st	1.56 ± 0.69 st	9.67 ± 1.32 t–v		0.64 ± 0.11 v	3.21 ± 0.43 op	4.25 ± 0.43 st	83.20 x
Ś43	17.88 ± 0.38 i–k	5.76 ± 0.32 gh	7.00 ± 0.67 e–g	662.2 ± 28.9 cd	16.76 ± 1.33 j–o		709.69 d–g	335.25 ± 3.12 fg	17.48 ± 1.37 fg	19.42 ± 0.23 o–s		4.57 ± 0.12 h–k	7.77 ± 1.65 g–j	8.85 ± 0.99 n–r	336.43 j–n
**Sample**	**Flavonols ^†^**								**Anthocyanins ^†^**						
	**Q-pentoside-hexoside**	**Q-3-galactoside**	**Q-3-glucoside**	**Q-3-rutinoside**	**Q-arabinoside**	**Q-rhamnoside**	**Q-penthoside rhamnoside**	**Other**	**Total**	**C-3-*O*-galactoside**	**C-3-*O*-glucoside**	**C-3-*O*-rutinoside**	**p-3-*O*-glucoside**	**Other**	**Total**
Ś1	2.49 ± 0.23 f–h	3.33 ± 0.11 o–t	10.73 ± 0.66 g	17.58 ± 2.10 e–h	5.88 ± 0.38 f–h	1.52 ± 0.43 jk	4.03 ± 0.21 de	7.76 ± 1.01 d	53.31 f–i	1.08 ± 0.23 o–u	3.48 ± 0.12 g	11.34 ± 0.32 e–h	1.91 ± 0.32 f–h	5.13 ± 0.54 cd	22.94 j–m
Ś2	1.09 ± 0.19 n–p	11.92 ± 1.11 hi	3.75 ± 0.43 m–p	8.59 ± 0.55 q–s	1.99 ± 0.63 p–u	0.84 ± 0.10 l–n	0.21 ± 0.02 p–s	nd u	28.38 n–r	3.87 ± 0.32 hi	1.22 ± 0.11 m–p	5.54 ± 0.21 q–s	0.65 ± 0.11 p–u	0.69 ± 0.09 uv	11.97 q–t
Ś3	1.22 ± 0.21 m–o	5.77 ± 0.21 l–o	4.78 ± 1.32 l–n	11.39 ± 1.77 k–q	3.56 ± 0.62 l–o	nd o	1.21 ± 0.14 j–n	0.87 ± 0.10 q–u	28.80 n–r	1.87 ± 0.44 l–o	1.55 ± 0-.13 l–n	7.35 ± 0.16 k–q	1.16 ± 0.32 l–o	1.07 ± 0.23 tu	13.00 q–s
Ś4	1.09 ± 0.32 n–p	3.72 ± 0.39 n–s	1.26 ± 0.37 s–v	10.60 ± 0.77 m–r	1.17 ± 0.38 s–v	0.64 ± 0.01 n	0.52 ± 0.19 o–s	2.41 ± 0.21 m–p	21.42 q–t	1.21 ± 0.12 n–t	0.41 ± 0.02 s–u	6.84 ± 0.43 m–r	0.38 ± 0.14 s–v	1.52 ± 0.32 p–t	10.35 r–v
Ś5	1.01 ± 0.11 n–p	4.62 ± 0.22 m–q	1.47 ± 0.34 r–v	12.48 ± 0.99 k–p	5.65 ± 0.29 f–h	nd o	nd s	1.51 ± 0.09 o–r	26.74 o–s	1.50 ± 0.11 m–q	0.48 ± 0.11 r–u	8.05 ± 0.37 k–p	1.83 ± 0.32 f–h	0.82 ± 0.23 t–v	12.68 q–t
Ś6	2.50 ± 0.17 f–h	7.01 ± 0.36 k–m	2.90 ± 0.66 o–s	20.59 ± 0.96 e	5.99 ± 0.76 fg	1.27 ± 0.22 j–l	2.05 ± 0.11 g	2.67 ± 0.47 l–n	44.97 i–l	2.28 ± 0.11 k–m	0.94 ± 0.11 o–s	13.29 ± 0.54 e	1.94 ± 0.11 fg	2.76 ± 0.12 j–m	21.20 k–n
Ś7	1.81 ± 0.11 j–l	9.13 ± 0.64 i–k	3.16 ± 0.28 n–q	7.71 ± 1.21 q–t	2.67 ± 0.99 n–r	1.70 ± 0.28 ij	0.69 ± 0.32 n–r	1.88 ± 0.54 n–q	28.75 n–r	2.96 ± 0.43 i–k	1.03 ± 0.23 n–q	4.97 ± 0.76 r–t	0.87 ± 0.21 n–r	1.97 ±0.43 n–q	11.80 q–t
Ś8	1.11 ± 0.09 n–p	2.64 ± 0.72 p–t	1.19 ± 0.46 t–v	8.99 ± 0.77 p–s	2.12 ± 0.10 p–u	0.73 ± 0.19 mn	1.00 ± 0.11 j–o	nd u	17.78 s–u	0.86 ± 0.15 p–u	0.39 ± 0.02 tu	5.80 ± 0.49 p–s	0.69 ± 0.13 p–u	0.92 ± 0.18 t–v	8.66 s–w
Ś9	2.54 ± 0.11 fg	1.05 ± 0.11 st	0.71 ± 0.02 v	13.87 ± 0.48 i–m	1.59 ± 0.24 r–u	nd o	3.41 ± 0.41 ef	3.37 ± 0.36 j–m	26.53 o–s	0.34 ± 0.43 tu	0.23 ± 0.12 u	8.95 ± 0.54 i–m	0.51 ± 0.04 r–u	3.03 ± 0.54 i–l	13.06 q–s
Ś10	0.91 ± 0.12 o–q	12.10 ± 0.43 h	3.91 ± 0.34 m–p	19.88 ± 0.54 ef	2.46 ± 0.19 o–s	3.62 ± 0.46 d	2.93 ± 0.38 f	1.42 ± 0.43 o–s	47.25 h–k	3.93 ± 0.16 h	1.27 ± 0.02 m–p	12.83 ± 0.28 ef	0.80 ± 0.06 o–s	2.88 ± 0.23 j–l	21.71 k–n
Ś11	1.24 ± 0.11 m–o	11.04 ± 0.77 h–j	4.41 ± 0.54 m–o	12.94 ± 0.48 j–o	3.80 ± 0.55 j–n	1.13 ± 0.21 k–m	0.85 ± 0.22 k–o	0.37 ± 0.19 tu	35.78 l–o	3.58 ± 0.32 h–j	1.43 ± 0.32 m–o	8.35 ± 0.87 j–o	1.23 ± 0.16 j–n	1.16 ± 0.33 r–u	15.76 o–q
Ś12	0.77 ± 0.21 p–r	20.09 ± 1.32 e–f	8.83 ± 0.84 ij	8.50 ± 0.77 q–s	3.09 ± 0.75 m–q	3.24 ± 0.32 de	0.60 ± 0.10 n–s	1.14 ± 0.63 q–t	46.28 h–l	6.52 ± 0.13 ef	2.87 ± 0.12 ij	5.49 ± 0.76 q–s	1.00 ± 0.11 m–q	1.87 ± 0.19 n–r	17.75 n–p
Ś13	nd t	1.57 ± 0.33 r–t	1.42 ± 0.39 r–v	13.63 ± 0.59 i–n	4.61 ± 0.39 h–l	1.46 ± 0.15 jk	4.12 ± 0.32 d	3.28 ± 0.49 j–m	30.09 n–r	0.51 ± 0.33 r–u	0.46 ± 0.02 r–u	8.79 i± 0.99 -n	1.50 ± 0.18 h–l	2.88 ± 0.12 j–l	14.14 p–r
Ś14	nd t	23.72 ± 0.54 d	10.56 ± 0.44 gh	14.86 ± 0.74 g–k	3.19 ± 0.76 m–p	2.15 ± 0.29 hi	0.73 ± 0.11 m–r	3.07 ± 0.29 k–m	58.28 e–g	7.70 ± 0.12 d	3.43 ± 0.23 gh	9.59 ± 0.27 g–k	1.03 ± 0.43 m–p	1.93 ± 0.32 n–q	23.68 j–l
Ś15	3.55 ± 0.56 d	1.12 ± 0.10 st	3.05 ± 0.93 o–r	7.58 ± 0.82 r–t	2.00 ± 0.39 p–u	0.86 ± 0.11 l–n	1.98 ± 0.29 gh	7.72 ± 0.73 d	27.85 o–s	0.36 ± 0.10 s–u	0.99 ± 0.11 o–r	4.89 0.56 r–t	0.65 ± 0.4 p–u	4.58 ± 0.43 d–f	11.47 q–u
Ś16	2.06 ± 0.29 h–k	6.53 ± 0.43 k–n	4.17 0.37 m–o	32.46 ± 0.73 cd	9.18 ± 3.66 cd	2.91 ± 0.29 ef	4.83 ± 0.43 c	3.62 ± 0.65 j–l	65.75 c–e	2.12 ± 0.12 k–n	1.35 ± 0.13 m–o	20.95 ± 1.11 cd	2.98 ± 0.23 cd	4.35 ± 0.65 e–g	31.75 d–f
Ś17	1.24 ± 0.33 m–o	8.42 ± 0.88 j–l	1.97 ± 0.43 q–v	18.29 ± 0.88 e–g	1.79 ± 0.65 q–u	1.38 ± 0.16 jk	1.43 ± 0.11 g–k	12.88 ± 1.32 b	47.41 h–k	2.73 ± 0.32 j–l	0.64 ± 0.02 q–u	11.81 ± 0.88 e–g	0.58 ± 0.07 q–u	5.50 ± 0.33 c	21.26 k–n
Ś18	1.41 ± 0.11 l–n	18.55 ± 1.72 fg	9.01 ± 1.21 hi	41.61 ± 0.34 a	11.77 ± 1.11 b	2.92 ± 0.32 ef	4.00 ± 0.88 de	7.53 ± 0.92 d	96.80 b	6.02 ± 0.25 fg	2.92 ± 0.12 hi	26.86 ± 0.77 a	3.82 ± 0.23 b	5.15 ± 0.54 cd	44.77 b
Ś19	3.63 ± 0.24 cd	4.37 ± 0.32 m–r	1.39 ± 0.46 s–v	16.72 ± 1.12 f–i	1.56 ± 0.21 r–u	nd o	0.76 ± 0.39 l–r	4.28 ± 0.19 g–j	32.70 m–p	1.42 ± 0.32 m–r	0.45 ± 0.07 s–u	10.79 ± 0.76 f–i	0.51 ± 0.11 r–u	2.81 ± 0.26 j–m	15.98 o–q
Ś20	1.38 ± 0.43 l–n	3.66 ± 0.37 n–t	2.05 ± 0.29 q–v	10.08 ± 1.08 n–r	5.26 ± 0.31 g–i	nd o	1.35 ± 0.52 i–m	14.59 ± 0.32 a	38.37 k–n	1.19 ± 0.15 n–t	0.66 ± 0.06 q–u	6.51 ± 1.00 n–r	1.71 ± 0.21 g–i	5.62 ± 0.41 c	15.69 o–q
Ś21	1.24 ± 0.48 m–o	0.75 ± 0.01 t	0.65 ± 0.08 v	7.60 ± 0.55 r–t	1.00 ± 0.11 t–v	nd o	1.36 ± 0.19 h–l	1.38 ± 0.21 p–t	13.98 tu	0.24 ± 0.02 u	0.21 ± 0.03 u	4.91 ± 0.10 r–t	0.32 ± 0.03 uv	1.29 ± 0.43 q–u	6.98 u–x
Ś22	4.45 ± 0.29 b	1.88 ± 0.21 q–t	7.26 ± 0.58 jk	18.78 ± 0.99 ef	24.12 ± 0.73 a	nd o	9.99 ± 0.42 a	5.31 ± 1.03 fg	71.79 cd	0.61 ± 0.4 q–u	2.36 ± 0.29 jk	12.12 ± 0.43 ef	7.83 ± 0.54 a	6.41 ± 0.73 b	29.32 f–h
Ś23	2.86 ± 0.54 ef	3.42 ± 0.11 o–t	2.51 ± 0.58 p–t	7.68 ± 0.29 r–t	3.60 ± 0.66 k–o	1.68 ± 0.11 j	1.54 ± 0.33 g–j	1.90 ± 0.11 n–q	25.19 p–s	1.11 ± 0.09 o–u	0.81 ± 0.08 p–t	4.96 ± 0.26 r–t	1.17 ± 0.27 k–o	2.59 ± 0.37 k–n	10.64 r–u
Ś24	nd t	4.90 ± 0.54 m–p	1.85 ± 0.29 q–v	4.66 ± 0.48 tu	1.11 ± 0.12 t–v	0.40 ± 0.12 no	0.19 ± 0.10 q–s	0.46 ± 0.19 s–u	13.58 tu	1.59 ± 0.12 m–p	0.60 ± 0.01 q–u	3.01 ± 0.77 tu	0.36 ± 0.02 t–v	0.34 ± 0.11 v	5.90 v–x
Ś25	0.42 ± 0.02 r–t	12.10 ± 0.99 h	17.35 ± 0.21 c	8.40 ± 0.29 q–s	7.73 ± 0.21 e	nd o	0.80 ± 0.22 l–q	6.37 ± 0.88 e	53.17 f–i	3.93 ± 0.32 h	5.63 ± 0.32 c	5.42 ± 0.29 q–s	2.51 ± 0.18 e	2.47 ± 0.22 l–o	19.95 l–o
Ś26	2.30 ± 0.20 g–i	4.64 ± 0.48 m–q	11.40 ± 0.42 fg	11.04 ± 0.54 l–r	4.11 ± 0.32 i–m	8.09 ± 0.11 a	10.06 ± 1.06 a	14.87 ± 1.32 a	66.50 c–e	1.50 ± 0.12 m–q	3.70 ± 0.12 fg	7.12 ± 0.88 l–r	1.33 ± 0.56 i–m	11.46 ± 0.67 a	25.12 h–k
Ś27	0.24 ± 0.11 st	1.13 ± 0.29 st	0.42 ± 0.01 v	6.02 ± 0.55 s–u	0.90 ± 0.13 uv	nd o	nd s	0.51 ± 0.01 r–u	9.21 u	0.37 ± 0.01 s–u	0.14 ± 0.01 u	3.89 ± 0.39 s–u	0.29 ± 0.02 uv	0.24 ± 0.04 v	4.92 wx
Ś28	nd t	3.51 ± 0.73 o–t	0.82 ± 0.01 uv	2.40 ± 0.64 u	0.15 ± 0.29 v	0.52 ± 0.05 n	0.15 ± 0.02 rs	0.07 ± 0.01 u	7.60 u	1.14 ± 0.12 o–u	0.26 ± 0.02 u	1.55 ± 0.44 u	0.05 ± 0.00 v	0.24 ± 0.01 v	3.24 x
Ś29	2.00 ± 0.10 i–k	16.40 ± 0.71 g	12.92 ± 0.39 ef	20.88 ± 0.49 e	11.97 ± 0.92 b	2.17 ± 0.23 h	1.15 ± 0.05 j–n	3.61 ± 0.12 j–l	71.09 cd	5.32 ± 0.43 g	4.19 ± 0.03 ef	13.48 ± 0.88 e	3.88 ± 0.34 b	2.90 ± 0.55 j–l	29.77 e–g
Ś30	nd t	9.98 ± 0.48 h–j	8.54 ± 0.32 ij	38.99 ± 2.73 ab	2.30 ± 0.13 o–t	nd o	1.85 ± 0.18 g–i	4.72 ± 0.32 f–i	66.37 c–e	3.24 ± 0.26 h–j	2.77 ± 0.12 ij	25.16 ± 0.66 ab	0.75 ± 0.06 o–t	2.13 ± 0.18 m–p	34.05 c–e
Ś31	1.30 ± 0.11 m–o	28.02 ± 0.49 c	11.02 ± 0.43 g	30.80 ± 0.38 d	7.97 ± 0.21 de	2.60 ± 0.32 f–h	1.60 ± 0.21 g–j	3.91 ± 0.12 h–k	87.21 b	9.09 ± 0.33 c	3.58 ± 0.65 g	19.88 ± 0.32 d	2.59 ± 0.03 de	3.05 ± 0.45 i–l	38.19 c
Ś32	4.12 ± 0.32 b	53.67 ± 0.39 a	20.34 ± 0.25 b	35.35 ± 1.88 bc	9.72 ± 0.45 c	nd o	1.56 ± 0.19 g–j	4.01 ± 0.54 h–k	128.77 a	17.42 ± 0.54 a	6.60 ± 0.66 b	22.82 ± 0.99 bc	3.16 ± 0.43 c	3.15 ± 0.45 i–l	53.14 a
Ś33	3.01 ± 0.10 e	18.67 ± 0.29 fg	6.16 ± 0.12 kl	16.67 ± 0.39 f–i	5.52 ± 0.44 f–h	2.71 ± 0.43 fg	1.37 ± 0.29 h–l	2.42 ± 0.65 m–o	56.53 e–h	6.06 ± 0.99 fg	2.00 ± 0.65 kl	10.76 ± 0.63 f–i	1.79 ± 0.25 f–h	3.08 ± 0.29 i–l	23.70 j–l
Ś34	0.36 ± 0.02 r–t	22.71 ± 0.83 de	14.22 ± 0.32 de	14.55 ± 1.32 h–l	4.67 ± 0.29 h–l	3.26 ± 0.29 de	0.82 ± 0.11 k–p	1.17 ± 0.29 q–t	61.75 d–f	7.37 ± 0.65 de	4.61 ± 0.55 de	9.39 ± 0.65 h–l	1.52 ± 0.43 g–l	1.82 ± 0.19 o–s	24.71 i–k
Ś35	2.93 ± 0.38 ef	4.11 ± 0.77 m–r	5.02 ± 0.86 lm	9.88 ± 0.74 o–r	1.97 ± 0.12 p–u	2.33 ± 0.33 gh	3.99 ± 0.32 de	0.61 ± 0.05 r–u	30.83 m–q	1.33 ± 0.36 n–r	1.63 ± 0.13 lm	6.37 ± 0.82 o–r	0.64 ± 0.52 p–u	3.20 ± 0.39 i–k	13.18 q–s
Ś36	1.65 ± 0.32 k–m	20.58 ± 1.43 ef	12.73 ± 0.19 ef	16.59 f–j	4.90 ± 0.74 g–k	6.20 ± 0.62 b	2.87 ± 0.17 f	1.46 ± 0.32 o–s	66.98 c–e	6.68 ± 0.43 ef	4.13 ± 0.43 ef	10.71± 0.69 f–j	1.59 ± 0.29 g–k	3.96 ± 0.38 f–h	27.06 g–j
Ś37	0.88 ± 0.06 o–q	16.55 ± 1.76 g	23.17 ± 0.32 a	12.56 k–p	11.71 ± 1.32 b	nd o	1.14 ± 0.21 j–o	9.52 ± 0.99 c	75.52 c	5.37 ± 0.67 g	7.52 ± 0.77 a	8.11 ± 0.73 k–p	3.80 ± 0.54 b	3.74 ± 0.19 g–i	28.54 f–i
Ś38	2.20 ± 0.19 g–j	1.76 ± 0.53 q–t	0.70 ± 0.11 v	29.52 d	5.07 ± 0.37 g–j	0.44 ± 0.05 no	4.58 ± 0.11 cd	4.89 ± 0.43 f–h	49.16 g–j	0.57 ± 0.09 q–u	0.23 ± 0.06 u	19.05 ± 0.88 d	1.65 ± 0.29 g–j	3.93 ± 0.44 f–h	25.42 g–k
Ś39	0.56 ± 0.03 q–s	39.72 ± 0.38 b	15.78 ± 0.98 cd	19.48 ef	6.75 ± 0.52 ef	5.26 ± 0.29 c	1.02 ± 0.32 j–o	3.81 ± 0.76 i–k	92.38 b	12.89 ± 0.87 b	5.12 ± 0.56 cd	12.57 ± 0.62 ef	2.19 ± 0.29 ef	3.46 ± 0.10 h–j	36.23 cd
Ś40	3.19 ± 0.11 de	3.96 ± 0.43 n–s	1.95 ± 0.28 q–v	14.52 h–l	1.79 ± 0.10 q–u	1.15 ± 0.18 k–m	1.37 ± 0.29 h–l	5.55 ± 0.39 ef	33.49 m–p	1.29 ± 0.88 n–s	0.63 ± 0.05 q–u	9.37 ± 0.99 h–l	0.58 ± 0.05 q–u	3.66 ± 0.29 g–i	15.53 o–q
Ś41	4.07 ± 0.32 bc	3.59 ± 0.29 o–t	4.17 ± 0.79 m–o	35.92 bc	12.42 ± 1.86 b	1.52 ± 0.55 jk	7.48 ± 0.72 b	4.23 ± 0.64 h–j	73.39 c	1.17 ± 0.43 o–u	1.35 ± 0.31 m–o	23.18 ± 1.34 bc	4.03 ± 0.29 b	5.61 ± 0.55 c	35.35 cd
Ś42	0.37 ± 0.05 r–t	3.75 ± 0.54 n–s	3.73 ± 0.54 m–p	5.74 s–u	3.14 ± 0.51 m–p	1.17 ± 0.43 k–m	0.73 ± 0.43 m–r	1.14 ± 0.49 q–t	19.79 r–t	1.22 ± 0.76 n–t	1.21 ± 0.23 m–p	3.71 ± 0.34 s–u	1.02 ± 0.38 m–p	1.11 ± 0.16 s–u	8.27 t–w
Ś43	6.91 ± 0.25 a	4.21 ± 0.77 m–r	2.47 ± 0.49 p–u	17.62 e–h	1.78 ± 1.63 r–u	1.42 j± 0.28 k	2.97 ± 0.49 f	3.30 ± 0.29 j–m	40.67 j–m	1.37 ± 0.56 m–r	0.80 ± 0.03 p–t	11.37 ± 1.93 e–h	0.58 ± 0.01 q–u	4.74 ± 0.39 de	18.86 m–o

^†^ means value of n = 3 independent repetition; nd—not detected; q-quercetin; c-cyanidin; p-peonidin; a, b, c,… values followed by the same letter within a column are not significantly different (*p* < 0.05; Tukey’s test).

**Table 4:** Biological activity as antioxidant capacity (ABTS^o+^, FRAP; mmoL Trolox/100 g d.w.) and antidiabetic, antiobesity, and anticholinergic activity (IC_50_ as mg/mL) of the tested *P. domestica* fruits.

Sample	Antioxidant Activity		Antidiabetic Acitvity ^†^		Antiobesity Activity ^†^	15-LOX ^†^	Anti-Aging Activity ^†^	
	ABTS^o+^	FRAP	α-Amylase	α-Glucosidase	Pancreatic lipase		AChE	BuChE
Ś1	5.38 ± 0.33 c–h	8.32 ± 0.29 g–l	nd r	2.92 ± 0.11 q	7.94 ± 0.02 a	9.27 ± 1.01 m–r	31.32 ± 0.72 j–m	48.60 ± 1.32 g–k
Ś2	6.97 ± 0.68 ab	11.71 ± 0.40 ab	nd r	nd r	2.98 ± 0.09 j–o	16.71 ± 0.43 e–f	37.68 ±1.03 f–k	56.33 ± 1.65 c–h
Ś3	4.53 ± 0.23 g–m	8.01 ± 0.60 h–m	nd r	2.06 ± 0.32 q–r	2.56 ± 0.11 n–t	11.58 ± 0.23 i–m	nd r	41.92 ± 2.33 j–n
Ś4	3.71 ± 0.51 m–o	6.12 ± 0.18 op	32.57 ± 2.15 g–k	1.84 ± 0.11 q–r	3.11 ± 0.06 i–o	9.54 ± 0.28 m–r	41.73 ± 1.64 e–h	52.73 ± 2.54 e–i
Ś5	3.63 ± 0.04 m–o	6.06 ± 0.26 op	31.70 ± 1.76 h–l	0.45 ± 0.31 r	4.23 ± 0.04 c–f	8.63 ± 0.54 n–s	42.71 ± 1.33 d–g	56.16 ± 1.42 c–h
Ś6	4.71 ± 0.59 e–l	8.45 ± 0.16 g–l	25.97 ± 2.41 m–o	nd r	5.60 ± 0.23 b	5.90 ± 0.99 t–v	25.19 ± 1.43 m–o	25.43 ± 1.76 p
Ś7	4.73 ± 0.61 e–l	8.75 ± 0.69 f–k	30.98 ± 2.51 i–m	nd r	3.70 ± 0.41 f–i	7.01 ± 0.29 r–u	38.85 ± 2.17 f–j	53.89 ± 2.54 d–i
Ś8	5.58 ± 0.09 c–f	9.47 ± 0.76 d–i	nd r	nd r	4.69 ± 0.23 cd	20.67 ± 0.76 c	36.70 ± 2.43 g–k	53.04 ± 1.65 e–i
Ś9	4.83 ± 0.30 e–l	8.90 ± 0.89 e–j	nd r	nd r	3.54 ± 0.24 g–k	6.59 ± 0.99 s–v	18.83 ± 1.43 op	32.22 ± 1.27 n–p
Ś10	3.16 ± 0.24 op	5.81 ± 0.30 op	30.70 ± 1.11 i–m	0.68 ± 0.40 r	3.58 ± 0.54 f–k	7.26 ± 0.69 q–u	27.87 ± 2.52 l–n	36.26 ± 2.54 no
Ś11	7.71 ± 0.47 a	13.28 ± 0.30 a	nd r	6.93 ± 0.65 m–o	3.20 ± 0.32 h–n	4.19 ± 0.67 v	40.97 ± 2.43 e–h	60.54 ± 1.52 b–f
Ś12	6.25 ± 0.64 bc	10.51 ± 0.22 b–e	nd r	0.19 ± 0.23 r	1.94 ± 0.26 t–w	8.28 ± 0.58 o–t	31.98 ± 1.11 i–m	37.15 ± 1.75 l–o
Ś13	6.59 ± 0.56 b	11.75 ± 0.29 ab	nd r	3.50 ± 1.12 p–q	2.50 ± 0.54 o–t	14.77 ± 0.76 f–h	42.29 ± 2.12 e–g	61.37 ± 1.99 b–e
Ś14	4.67 ± 0.47 f–l	9.08 ± 0.43 d–i	29.78 ± 2.88 i–m	10.44 ± 1.01 i–k	2.66 ± 0.83 n–s	10.73 ± 0.99 k–o	39.43 ± 3.21 f–i	51.88 ± 2.72 e–i
Ś15	2.45 ± 0.24 pq	3.57 ± 0.30 q	42.34 ± 2.71 c–e	14.98 ± 2.43 d–f	4.82 ± 0.88 c	18.65 ± 0.73 c–e	61.72 ± 3.41 a	64.72 ± 2.41 bc
Ś16	4.18 ± 0.33 j–n	7.13 ± 0.16 k–p	5.24 ± 0.31 r	5.14 ± 0.32 o–p	2.77 ± 0.34 l–q	11.40 ± 0.67 j–m	33.11 ± 2.12 i–l	46.90 ± 1.99 h–l
Ś17	3.10 ± 0.20 o–q	5.59 ± 0.08 p	47.44 ± 2.54 c	19.72 ± 2.11 b	3.63 ± 0.88 f–j	24.67 ±0.78 b	44.33 ± 2.16 c–f	56.10 ± 1.62 c–h
Ś18	5.46 ± 0.45 c–g	9.85 ± 0.38 c–g	27.15 ± 1.11 k–n	8.56 ± 0.67 k–m	2.09 ± 0.75 r–u	12.40 ± 0.65 h–k	20.62 ± 2.11 n–p	39.34 ± 1.61 k–n
Ś19	4.94 ± 0.25 e–k	8.88 ± 0.18 e–j	21.89 ± 1.43 n–p	1.89 ± 0.12 qr	1.65 ± 0.54 u–v	9.94 ± 0.48 k–p	7.62 ± 0.54 q	29.06 ± 2.84 op
Ś20	6.07 ± 0.62 b–d	11.33 ± 0.28 bc	32.68 ± 2.52 g–j	11.75 ± 1.12 g–i	2.68 ± 0.78 n–r	9.83 ± 0.99 k–q	33.03 ± 2.54 i–l	48.11 ± 2.91 g–k
Ś21	3.25 ± 0.12 n–p	6.88 ± 0.24 l–p	53.57 ± 1.54 b	11.49 ± 0.32 g–i	3.44 ± 0.45 g–l	17.07 ± 0.69 d–f	50.77 ± 1.12 bc	48.76 ± 1.63 g–k
Ś22	2.64 ± 0.4 pq	6.06 ± 0.17 op	37.69 ± 1.11 e–g	11.67 ± 1.01 g–i	1.40 ± 0.56 vw	8.40 ± 0.67 o–t	30.36 ± 1.25 k–m	28.93 ± 1.45 op
Ś23	5.04 ± 0.35 e–j	10.60 ± 0.65 b–d	28.23 ± 0.65 j–m	7.68 ± 0.13 l–n	2.76 ± 0.54 m–r	12.30 ± 0.73 h–l	41.97 ± 1.23 e–g	51.07 ± 1.63 f–j
Ś24	5.59 ± 0.89 c–f	9.36 ± 0.14 d–i	nd r	15.65 ± 1.23 d	3.53 ± 0.65 g–k	6.09 ± 0.56 s–v	38.77 ± 1.32 f–j	57.01 ± 1.52 c–g
Ś25	4.84 ± 0.20 e–k	8.68 ± 0.12 f–k	19.79 ± 1.52 pq	10.31 ± 1.16 i–k	4.45 ± 0.48 c–e	4.80 ± 0.48 uv	50.87 ±1.45 bc	65.08 ± 2.72 bc
Ś26	3.18 ± 0.25 op	6.48 ± 0.31 m–p	38.90 ± 1.43 d–f	19.62 ± 0.21 b	2.79 ± 0.76 l–p	12.30 ± 0.99 h–l	47.47 ± 2.15 b–e	47.94 ± 1.99 g–p
Ś27	5.43 ± 0.70 c–h	10.36 ± 0.36 b–f	17.18 ± 1.59 pq	8.66 ± 0.54 km	3.38 ± 0.41 g–m	14.01 ± 0.65 g–i	21.26 ± 2.43 no	36.64 ± 2.61 m–o
Ś28	5.08 ± 0.58 e–j	8.85 ± 0.35 e–j	nd r	13.15 ± 1.43 f–h	2.18 ± 0.54 p–u	7.75 ± 0.46 p–t	39.41 ± 2.55 f–i	70.26 ± 2.71 ab
Ś29	4.52 ± 0.50 h–m	8.63 ± 0.29 g–k	21.13 ± 0.54 op	9.59 ± 1.00 i–l	1.98 ± 0.43 t–v	10.51 ± 0.59 k–o	13.32 ± 2.43 pq	15.60 ± 2.61 q
Ś30	4.42 ± 0.29 i–m	7.81 ± 0.14 i–n	32.67 ± 2.41 g–j	13.36 ± 1.01 e–g	2.25 ± 0.32 p–u	10.41 ± 0.68 k–o	18.07 ± 1.65 op	35.52 ± 1.11 no
Ś31	2.48 ± 0.11 pq	3.34 ± 0.08 q	30.97 ± 2.54 i–m	14.50 ± 2.11 d–f	2.94 ± 0.53 k–o	19.41 ± 0.69 c–d	40.77 ± 1.67 e–h	51.43 ± 2.61 f–j
Ś32	4.90 ± 0.59 e–k	11.26 ± 0.44 bc	37.02 ± 1.11 e–h	16.27 ± 0.43 cd	1.39 ± 0.75 vw	9.79 ± 0.62 l–q	35.38 ± 1.79 g–l	35.29 ± 2.88 no
Ś33	4.01 ± 0.18 k–o	6.23 ± 0.19 n–p	nd r	10.19 ± 0.41 i–k	1.99 ± 0.65 s–v	32.67 ± 0.59 a	34.26 ± 2.43 h–l	50.49 ± 2.99 g–i
Ś34	3.90 ± 0.56 l–o	6.94 ± 0.47 l–p	2.63 ± 0.11 r	6.01 ±0.32 n–o	3.81 ± 0.47 e–h	10.26 ± 0.49 k–p	50.13 ± 2.11 b–d	64.12 ± 3.99 bc
Ś35	3.12 ± 0.43 o–q	6.54 ± 0.21 m–p	26.44 ± 0.22 l–o	13.35 ±0.78 e–g	0.44 ± 0.65 x	6.00 ± 0.59 t–v	19.65 ± 2.43 op	nd r
Ś36	5.64 ± 0.36 c–e	9.75 ± 0.06 c–g	nd r	15.50 ± 1.32 de	4.89 ± 0.56 c	4.98 ± 0.59 uv	61.82 ± 1.11 a	75.73 ± 3.11 a
Ś37	4.88 ± 0.56 e–k	9.53 ± 0.46 d–h	19.47 ± 0.76 pq	15.97 ± 1.54 d	3.00 ± 0.47 j–o	18.52 ± 0.50 c–e	41.82 ± 2.12 e–h	62.91 ± 1.64 b–d
Ś38	5.27 ± 0.34 d–i	9.68 ± 0.28 c–h	15.26 ± 1.43 q	11.07 ± 2.13 h–j	4.77 ± 0.57 c	16.81 ± 0.47 ef	52.47 ± 1.11 b	53.65 ± 2.61 d–i
Ś39	6.08 ± 0.67 b–d	10.36 ± 0.58 b–f	nd r	9.15 ± 2.11 j–l	1.28 ± 0.54 w	15.59 ± 0.38 fg	37.65 ± 2.65 f–k	32.30 ± 2.75 n–p
Ś40	3.35 ± 0.07 n–p	6.26 ± 0.22 n–p	43.62 ± 3.16 cd	18.39 ± 3.01 bc	2.10 ± 0.54 q–u	13.49 ± 0.56 g–j	50.83 ± 2.43 bc	46.22 ± 1.75 i–m
Ś41	2.20 ± 0.24 q	3.25 ± 0.05 q	61.53 ± 2.47 a	24.07 ± 2.74 a	4.03 ± 0.38 d–g	16.99 ± 0.49 d–f	45.20 ± 2.13 b–f	60.45 ± 2.94 b–f
Ś42	3.61 ± 0.23 m–o	7.35 ± 0.06 j–o	40.36 ± 1.11 de	16.33 ± 2.01 cd	3.95 ± 0.58 e–g	11.08 ± 0.39 j–n	45.15 ± 2.43 b–f	52.71 ± 1.59 e–i
Ś43	5.55 ± 0.71 c–f	9.19 ± 0.47 d–i	34.58 ± 3.54 f–i	nd r	4.45 ± 0.54 c–e	5.81 ± 0.59 t–v	44.46 ± 0.65 c–f	61.68 ± 2.01 b–e

^†^ mean value of n = 3 independent repetition; nd—not detected. a, b, c,… values followed by the same letter within a column are not significantly different (*p* < 0.05; Tukey’s test).

## Data Availability

All related data and methods are presented in this paper. Additional inquiries should be addressed to the corresponding authors.
